# Model incorporating multiple diffusion MRI features: development and validation of a radiomics-based model to predict adult-type diffuse gliomas grade

**DOI:** 10.1007/s00330-023-09861-0

**Published:** 2023-07-13

**Authors:** Peng Wang, Shenghui Xie, Qiong Wu, Lixin Weng, Zhiyue Hao, Pengxuan Yuan, Chi Zhang, Weilin Gao, Shaoyu Wang, Huapeng Zhang, Yang Song, Jinlong He, Yang Gao

**Affiliations:** 1 Department of Radiology, Affiliated Hospital of Inner Mongolia Medical University, Hohhot, 010059 China; 2 Inner Mongolia Medical University, Hohhot, 010110 China; 3 Department of Pathology, Affiliated Hospital of Inner Mongolia Medical University, Hohhot, 010059 China; 4 MR Scientific Marketing, Siemens Healthineers, Shanghai, 201318 China

**Keywords:** Brain, Glioma, Neoplasm grading, Diffusion magnetic resonance imaging, Biomarkers

## Abstract

**Objectives:**

To develop and validate a radiomics-based model (ADGGIP) for predicting adult-type diffuse gliomas (ADG) grade by combining multiple diffusion modalities and clinical and imaging morphologic features.

**Methods:**

In this prospective study, we recruited 103 participants diagnosed with ADG and collected their preoperative conventional MRI and multiple diffusion imaging (diffusion tensor imaging, diffusion kurtosis imaging, neurite orientation dispersion and density imaging, and mean apparent propagator diffusion-MRI) data in our hospital, as well as clinical information. Radiomic features of the diffusion images and clinical information and morphological data from the radiological reports were extracted, and multiple pipelines were used to construct the optimal model. Model validation was performed through a time-independent validation cohort. ROC curves were used to evaluate model performance. The clinical benefit was determined by decision curve analysis.

**Results:**

From June 2018 to May 2021, 72 participants were recruited for the training cohort. Between June 2021 and February 2022, 31 participants were enrolled in the prospective validation cohort. In the training cohort (AUC 0.958), internal validation cohort (0.942), and prospective validation cohort (0.880), ADGGIP had good accuracy in predicting ADG grade. ADGGIP was also significantly better than the single-modality prediction model (AUC 0.860) and clinical imaging morphology model (0.841) (all *p* < .01) in the prospective validation cohort. When the threshold probability was greater than 5%, ADGGIP provided the greatest net benefit.

**Conclusion:**

ADGGIP, which is based on advanced diffusion modalities, can predict the grade of ADG with high accuracy and robustness and can help improve clinical decision-making.

**Clinical relevance statement:**

Integrated multi-modal predictive modeling is beneficial for early detection and treatment planning of adult-type diffuse gliomas, as well as for investigating the genuine clinical significance of biomarkers.

**Key Points:**

*• Integrated model exhibits the highest performance and stability.*

*• When the threshold is greater than 5%, the integrated model has the greatest net benefit.*

*• The advanced diffusion models do not demonstrate better performance than the simple technology.*

**Supplementary information:**

The online version contains supplementary material available at 10.1007/s00330-023-09861-0.

## Introduction

Adult-type diffuse gliomas (ADGs) are the most common subtype of glioma [1]. Because of the large nuclear heterogeneity of tumor cells, the biological behavior of ADG features invasive growth, and clinically, the tumors are characterized by high disability-adjusted life years. The outcome of the tumor patient depends on a number of genetic traits as well as the histological grade [2]. Additionally, hereditary traits may play a bigger role in the overall development of the disease. However, the 2021 World Health Organization (WHO) Classification of Tumors of the Central Nervous System (CNS) successfully combines the two, and the resulting WHO grade may be a superior clinical biological indicator than an independent predictor (e.g., isocitrate dehydrogenase) in earlier studies [3]. With the continuous exploration and discovery of tumor growth mechanisms and new genetic biomarkers, some new treatment methods have shown great potential, such as immunotherapy [4]. Such advancements are expected to improve long-term survival and quality of life for the subset of patients who cannot be treated surgically, such as those with brainstem or multifocal lesions. Therefore, there is an urgent need for a reliable and noninvasive method to predict ADG grade to help develop precision medicine protocols.

MRI has long been the main method for the diagnosis of brain tumors. Moreover, with the development of various advanced imaging technologies, the clinical application of MRI technology is highly promising [5]. However, such biomarkers have not been widely used in clinical practice due to their complex theoretical composition and lack of effective prospective validation. Previous studies [6–9] have shown that advanced diffusion technologies can help to identify biological tumor markers, evaluate the damage of fiber tracts, or display the microstructure in the brain. We believe that multiple diffusion technologies can provide information reflecting pathological features, which can complement tumor heterogeneity and existing clinical applications.

Radiomics has made rapid progress in its applications in the medical field [10], especially in supporting decisions to enable machine-assisted diagnosis and prognosis assessments [11]. Previous studies [12] have shown that radiomic-based methods can be used to analyze the genetic characteristics of gliomas with improved performance. However, these studies were usually limited to conventional MRI, did not involve advanced imaging modalities [13] such as mean apparent propagation diffusion-MRI (MAP-MRI), or had low reproducibility due to the lack of prospective validation. Thus, we hypothesized that advanced diffusion technologies can be used for ADG grade prediction. To our knowledge, no similar study has been published.

In this study, we aimed to develop and validate a new integrated model for predicting ADG grade (adult-type diffuse gliomas grade integrated prediction model (ADGGIP)) by combining radiomic, clinical, and imaging morphological features. Additionally, the differences between the advanced diffusion technology and the simple technology were compared.

## Materials and methods

We conducted a prospective study in accordance with the Declaration of Helsinki. The ethics committee of our hospital approved the study protocol (No. 2022038), and all participants signed informed consent forms prior to enrollment in the cohort.

### Participants and clinical data

**Fig. 1:**
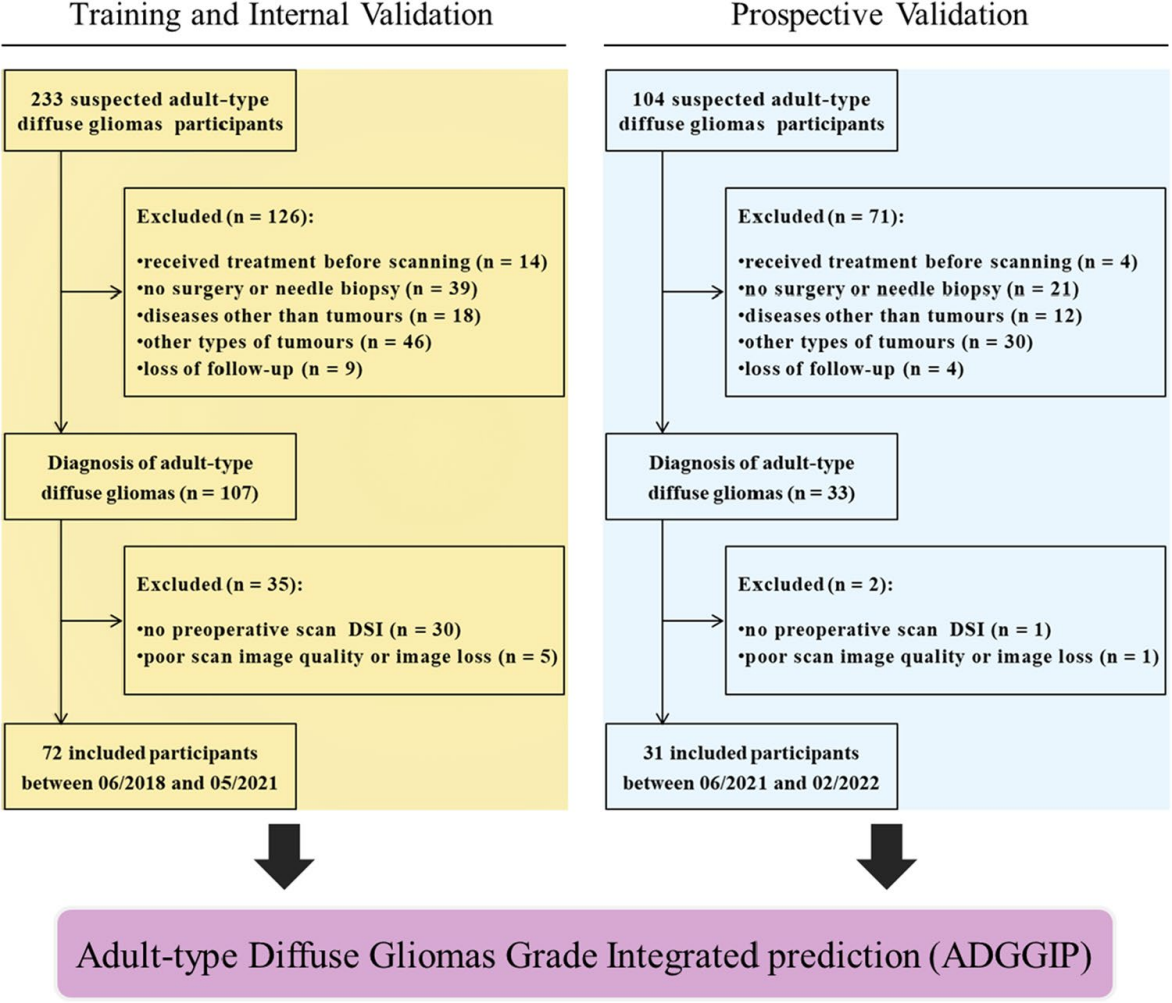
Recruitment pathway for study participants. Based on the date of enrolment, participants were divided into various datasets. The ADGGIP was developed and validated before being formed. DSI, diffusion spectrum magnetic resonance imaging

In this study, we prospectively and consecutively enrolled participants who visited our hospital from June 2018 to February 2022. All participants were suspected of ADG due to clinical symptoms or previous imaging reports. Then, all participants underwent preoperative conventional MRI (T2, T1, T2-FLAIR, and contrast-enhanced T1) and diffusion imaging (DWI and diffusion spectrum magnetic resonance imaging [DSI]), and surgery was performed within 3 months after the scan to obtain sufficient pathological tissue for the diagnosis of ADG (in accordance with the 2021 WHO Classification of Tumors of the CNS). Most participants received only general symptomatic care, such decreasing cranial pressure, between the MRI scan and surgery. For detailed scanning equipment, scanning parameters and pathological diagnosis information, see pages 1–2 in the Appendix and Supplementary Table S1. Certain participants were excluded based on the exclusion criteria (Fig. 1). Finally, 103 participants with ADG (mean age, 52 years; range, 21–77; 54 [52%] male) were enrolled. Because this was a single-center study, the time-validation method was used to validate the model [14], and participants were assigned to the training and prospective validation cohorts according to enrollment time. Seventy-two participants between June 2018 and May 2021 were assigned to the training cohort, including 38 low-grade glioma (LGG, CNS WHO Grades 2–3) participants and 34 high-grade glioma (HGG, CNS WHO Grade 4) participants. Thirty-one participants between June 2021 and February 2022 were assigned to the prospective validation cohort, including 8 participants with LGG and 23 participants with HGG.

### Model development and validation

We developed and compared the performance and stability of various models, with the aim of comparing the abilities of the advanced diffusion technology and the simple technology in identifying ADG grade and to establish a clinically applicable and more complete prediction model.

**Fig. 2:**
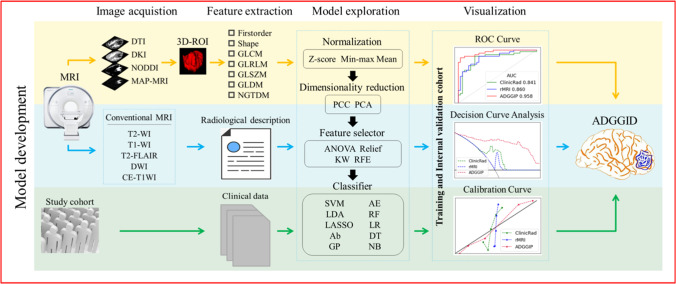
Workflow of the study. Information from preprocessed multilayer diffusion models, raw conventional MRI scans, and clinical features of the study cohort were collected and analyzed to summarize the underlying feature matrices that could be used to build the machine learning models. Model construction was performed using FeAture Explorer (V0.5.2), in which a variety of modeling approaches were tried. The mean receiver operating characteristic (ROC) curves, decision analysis curves and calibration curves were used to construct an integrated diagnostic model (ADGGID). DTI, diffusion tensor imaging; DKI, diffusion kurtosis imaging; NODDI, neurite orientation dispersion and density imaging; MAP-MRI, mean apparent propagation diffusion-MRI; ROI, region of interest; GLCM, gray level co-occurrence matrix; GLRLM, gray level run length matrix; GLSZM, gray level size zone matrix; GLDM, gray level dependence matrix; NGTDM, neighborhood gray tone difference matrix; PCC, Pearson correlation coefficient; PCA, principal component analysis; ANOVA, analysis of variance; KW, Kruskal-Wallis; RFE, recursive feature elimination; SVM, support vector machine; AE, auto-encoder; LDA, linear discriminant analysis; RF, random forest; Lasso, logistic regression via least absolute shrinkage and selection operator; LR, logistic regression; Ab, ada-boost; DT, decision tree; GP, Gaussian process; NB, native Bayes; ADGGIP, adult-type diffuse gliomas grade integrated prediction model

After image preprocessing and region of interest selection (Appendix, p. 3–4), feature extraction and model building were performed using FeAture Explorer (FAE v0.5.2, https://github.com/salan668/FAE) [15]. A total of 2782 radiomic features were extracted from the MRI data of 3 advanced technologies (including diffusion kurtosis imaging [DKI], neurite orientation dispersion and density imaging [NODDI], and MAP-MRI), 1 simple technology (diffusion tensor imaging [DTI]) and B0 map (Appendix p. 4–5, Supplementary Figure S1 and Table S2). In addition, sex and age were extracted as clinical features, and 9 morphological features (necrosis, cystic, calcification, hemorrhage, tumor enhancement pattern, location, side, clarity of the solid tumor boundary, and edema) were extracted from the imaging reports (Appendix p. 5–6). Then, multiple pipeline combinations were considered during model development, including 3 feature normalization methods (mean, min–max and *Z* score normalization), 2 data dimensionality reduction methods (principal component analysis and Pearson correlation coefficients) (Supplementary Figure S2 and S3), 4 feature selection methods (analysis of variance, recursive feature elimination, Kruskal‒Wallis, and Relief), and 10 classifiers (linear [logistic regression, logistic regression via least absolute shrinkage and selection operator, linear discriminant analysis, and support vector machine] and nonlinear [autoencoder, decision tree, random forest, ada-boost, Gaussian process, and naïve Bayes]), for a total of 240 pipelines (Appendix p. 7–9). Model evaluation was performed using an internal validation cohort (by leave-one-out cross-validation) and an independent validation cohort. The flowchart of this study is shown in Fig. 2.

Finally, a total of 9 diagnostic models were established, including 5 single-modality prediction models (B0, DTI, DKI, NODDI, and MAP-MRI models) based on single diffusion technology, 1 fusion prediction model incorporating all diffusion technologies (radiomics MRI [rMRI]), 1 prediction model with clinical and imaging morphological features (ClinicRad), and 2 multimodal prediction models (the single-modality model with the highest diagnostic performance in the prospective validation cohort and the more theoretically relevant prediction model incorporating multiple diffusion features were selected) (incorporating clinical factors, radiologists’ interpretations, and DTI or rMRI data [CliRadDTI/ADGGIP] to predict ADG grade) (Supplementary Figure S4).

### Statistical analysis

The performance of the model in predicting ADG grade was evaluated with the receiver operating characteristic curve. The 95% confidence intervals of the area under the curve (AUC) were generated by bootstrap with 1000 samples. We used the DeLong test, net reclassification improvement and integrated discrimination improvement to compare the performances of different models. Deviations between the model and the real results were visualized by calibration curves and quantified by the Brier score. Decision curve analysis was used to compare the net benefits of different models at different threshold probabilities to increase the possibility of practical application in clinical practice.

Quantitative data are expressed as the mean ± standard deviation. Student’s *t* test was used to compare age, and the *χ*^2^ test, Fisher test, or Mann‒Whitney *U* test were used to compare categorical variables. All statistical analyses were two-sided, and *p* < 0.05 was considered statistically significant. All statistical analyses were performed using SPSS (version 24.0); R with the irr, pROC, and PredictABEL packages installed (version 4.1.2); or Python (version 3.9.12) and Scikit-Learn (version 0.24.2). Sample size and power calculations are shown in the additional materials (Appendix p. 9–10).

## Results

### Participant characteristics

The baseline characteristics of all participants are summarized in Table 1 and Supplementary Table S3. There were no significant differences in baseline characteristics between the training and prospective validation cohorts, except for WHO grade (*p* = 0.012), tumor location (0.011), and side of the tumor (0.046). We attribute this to the increasing incidence of glioblastoma and to new WHO criteria that have elevated some histological grades based on genetic characteristics. However, the imbalanced classification still reflects the severity of gliomas (2/3 of ADG are HGG) in the real world.

**Table 1:** Participants characteristics

Variable	Training and internal validation cohort (*n* = 72)			Prospective validation cohort (*n* = 31)		
	LGG (*n* = 38)	HGG (*n* = 34)	*p* value	LGG (*n* = 8)	HGG (*n* = 23)	*p* value
Age (years)	46.21 ± 10.08	57.88 ± 10.35	< .001	49.38 ± 14.30	53.65 ± 13.12	.444
Sex			.637			.412
Male	20/38 (52.63%)	16/34 (47.06%)		6/8 (75.00%)	12/23 (52.17%)	
Female	18/38 (47.37%)	18/34 (52.94%)		2/8 (25.00%)	11/23 (47.83%)	
Necrosis			.001			.027
Present	22/38 (57.89%)	31/34 (91.18%)		3/8 (37.50%)	19/23 (82.61%)	
Absent	16/38 (42.11%)	3/34 (8.82%)		5/8 (62.50%)	4/23 (17.39%)	
Hemorrhage			.001			.660
Present	22/38 (57.89%)	31/34 (91.18%)		5/8 (62.50%)	17/23 (73.91%)	
Absent	16/38 (42.11%)	3/34 (8.82%)		3/8 (37.50%)	6/23 (26.09%)	
Calcification			.043			.335
Present	12/38 (31.58%)	4/34 (11.76%)		3/8 (37.50%)	4/23 (17.39%)	
Absent	26/38 (68.42%)	30/34 (88.24%)		5/8 (62.50%)	19/23 (82.61%)	
Cyst or cysts			.592			.999
Present	32/38 (84.21%)	31/34 (91.18%)		7/8 (87.50%)	21/23 (91.30%)	
Absent	6/38 (15.79%)	3/34 (8.82%)		1/8 (12.50%)	2/23 (8.70%)	
Edema (≤ 1.5 cm)			< .001			.412
Yes	29/38 (76.32%)	12/34 (35.29%)		3 (37.50%)	14 (60.87%)	
No	9/38 (23.68%)	22/34 (64.71%)		5 (62.50%)	9 (39.13%)	
Tumor borders			.979			.999
Sharp	18/38 (47.37%)	16/34 (47.06%)		5/8 (62.50%)	13/23 (56.52%)	
Blurry	20/38 (52.63%)	18/34 (52.94%)		3/8 (37.50%)	10/23 (43.48%)	
Tumor location category			< .001			.034
Frontal or insula	32/38 (84.21%)	12/34 (35.29%)		5 (62.50%)	4 (17.39%)	
Other	4/38 (10.53%)	20/34 (58.82%)		2 (25.00%)	16 (69.57%)	
Basal nucleus or corpus callosum	2/38 (5.26%)	2/34 (5.88%)		1 (12.50%)	3 (13.04%)	
Side			.083			.999
Left	18/38 (47.37%)	23/34 (67.65%)		3 (37.50%)	8 (34.78%)	
Right	20/38 (52.63%)	11/34 (32.35%)		5 (62.50%)	15 (65.22%)	
Enhancement category			< .001			.034
Patchy enhancing	15/38 (39.47%)	2/34 (5.88%)		5/8 (62.50%)	3/23 (13.04%)	
Ringlike enhancing	12/38 (31.58%)	31/34 (91.18%)		3/8 (37.50%)	19/23 (82.61%)	
Nonenhancing	11/38 (28.95%)	1/34 (2.94%)		0/8 (0.00%)	1/23 (4.35%)	

Data are the mean ± standard deviation or *n*/*N* (%), where *N* is the total number of patients with available data. *p* values were calculated with the chi-square test, Fisher’s test, Student’s *t* test, and Mann‒Whitney *U* test

*LGG* low-grade glioma, *HGG* high-grade glioma

In the training cohort, only sex (*p* = 0.637), cystic changes (0.592), tumor boundary (0.979), and side of the tumor (0.083) showed no significant differences between the LGG and HGG groups. In the prospective validation cohort, there were no significant differences in baseline characteristics between the LGG and HGG groups except for necrosis (*p* = 0.027), tumor location (0.034), and enhancement mode (0.034).

### Feature selection and pipeline

ADGGIP was established using a support vector machine. Unlike the single-modality radiomics model, the multimodal model incorporates clinical and imaging morphological features. ADGGIP was composed of four features, including one radiomics feature and three clinical features, among which the radiomics feature was the prediction probability of settlement on rMRI, and the three clinical features were calcification, tumor location, and edema; the contributions of the four features were 1.41, 0.94, 0.82, and 0.41, respectively. The radiomics feature had the highest correlation with tumor grade. Edema had the lowest correlation with tumor grade (Supplementary Figure S5). rMRI was composed of four radiomics features, all of which were first-order features. The energy and median values of the DTI fractional anisotropy and MAP non-Gaussianity axial are included, respectively. The feature distribution is shown in Supplementary Figure S6. The detailed constituent factors and pipelines of all models are shown in Table 2.

**Table 2:** Selected features for model construction

Feature origin (*N*)^a^	Pipeline^c^ (normalization/dimension reduction/feature selector/classifier)	Feature names
B0^b^ features (*N* = 5)	*Z* Score/PCC/KW/AE	gldm_DependenceVariance
		glszm_LargeAreaHighGrayLevelEmphasis
		ngtdm_Busyness
		ngtdm_Coarseness
		shape_MajorAxisLength
DTI^b^ features (*N* = 3)	MinMax/PCC/RFE/NB	AD_ firstorder_Minimum
		FA_ firstorder_Energy
		FA_ firstorder_Median
DKI^b^ features (*N* = 7)	*Z* Score/PCA/RFE/AE	PCA_feature_3
		PCA_feature_9
		PCA_feature_13
		PCA_feature_15
		PCA_feature_21
		PCA_feature_39
		PCA_feature_72
NODDI^b^ features (*N* = 4)	MinMax/PCA/Rel/LR-Lasso	PCA_feature_18
		PCA_feature_27
		PCA_feature_25
		PCA_feature_68
MAP-MRI^b^ features (*N* = 6)	*Z* Score/PCA/RFE/AE	PCA_feature_1
		PCA_feature_3
		PCA_feature_4
		PCA_feature_19
		PCA_feature_22
		PCA_feature_28
rMRI^b^ features (*N* = 4)	MinMax/PCC/RFE/AE	DTI_FA_ firstorder_Energy
		DTI_FA_ firstorder_Median
		MAP_NGAx_ firstorder_Energy
		MAP_NGAx_ firstorder_Median
ClinicRad^b^ features (*N* = 5)	*Z* Score/PCC/RFE/AE	Age
		Necrosis
		Hemorrhage
		Calcification
		Tumor location category
CliRadDTI^b^ features (*N* = 8)	MinMax/PCC/ANOVA/AE	Rad score-DTI^d^
		Age
		Necrosis
		Hemorrhage
		Calcification
		Edema
		Tumor location category
		Side
ADGGIP^b^ features (*N* = 4)	*Z* Score/PCC/Rel/SVM	Rad score-rMRI^e^
		Calcification
		Tumor location category
		Edema

Eight different modeling approaches were tried in the study, and a comprehensive evaluation analysis was carried out

^a^The total number of features in a distinct group

^b^We included 9 different modeling approaches for identifying adult-type diffuse gliomas grades

^c^The processing of valid data features for modeling was called a pipeline

^d,^^e^The prediction results of the DTI or rMRI model. The resulting predictive features were defined as a new feature matrix for further development of the multimodal prediction model

*B0* diffusion b0 parameter diagram, *DTI* diffusion tensor imaging, *DKI* diffusion kurtosis imaging, *NODDI* neurite orientation dispersion and density imaging, *MAP-MRI* mean apparent propagation diffusion-MRI, *rMRI* radiomics MRI, *ClinicRad* model incorporating clinical factors and interpretations from radiologists, *CliRadDTI *model incorporating clinical factors, radiologist interpretations and DTI data, *ADGGIP* adult-type diffuse gliomas grade integrated prediction model, *PCC* Pearson correlation coefficient, *PCA* principal component analysis, *KW* Kruskal‒Wallis, *RFE* recursive feature elimination, *Rel* relief, *ANOVA* analysis of variance, *AE* autoencoder, *NB* native Bayes, *LR-Lasso* logistic regression via least absolute shrinkage and selection operator, *SVM* support vector machine

Briefly, ADGGIP was constructed as an integrated prediction model by integrating radiomic, clinical, and imaging morphological features of the training cohort and was considered as the optimal model in this study. The ADGGIP provided a probabilistic forecast of ADG grade (ranging from 0 to 1), and the probability of LGG inversely correlated with the value of the anticipated probability output. Then, the result was artificially converted into a binary prediction, either LGG or HGG, where the threshold value relied on the maximum Youden index.

### Development, performance, and validation of prediction models

**Fig. 3:**
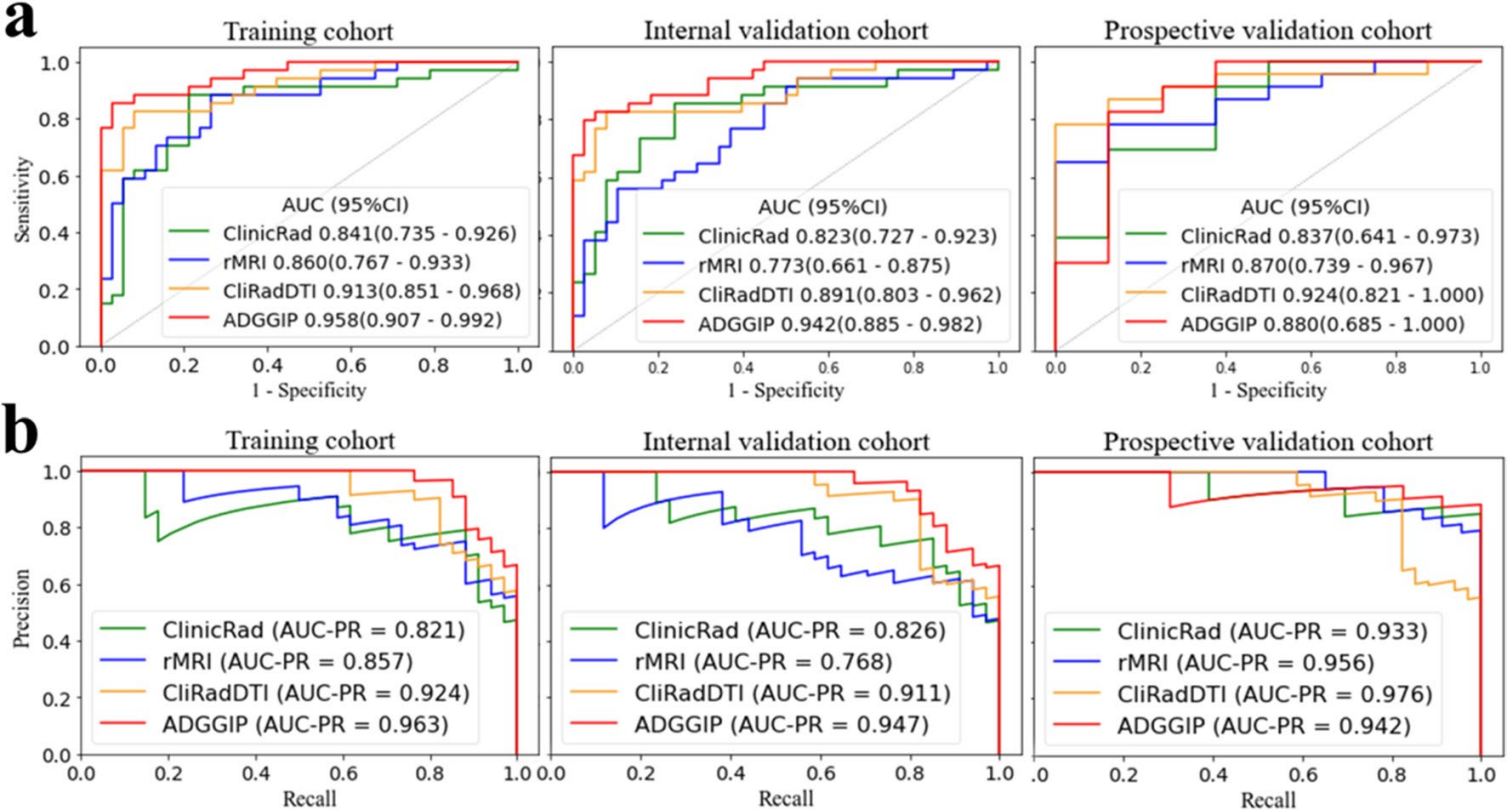
ROC curves (**a**) and PR curves (**b**) of ADGGIP and other integrated prediction models in the training and validation cohorts. ClinicRad, model incorporating clinical factors and interpretations from radiologists; rMRI, radiomics MRI; CliRadDTI, model incorporating clinical factors, radiologist interpretations and DTI data; ADGGIP, adult-type diffuse gliomas grade integrated prediction model

ADGGIP showed the strongest ability to discriminate tumor grades in the training cohort (AUC 0.958 [95% CI 0.907–0.992]) and internal validation cohort (0.942 [95% CI 0.885–0.982]) (Table 3 and Fig. 3). ADGGIP also showed superior performance in predicting tumor grade in the prospective validation cohort. Among 8 participants with LGG predicted by ADGGIP, 7 (87.5%) were pathologically confirmed to have LGG. In addition, among 23 HGG participants predicted by ADGGIP, 19 participants (82.6%) were confirmed to have HGG by pathology (Supplementary Figure S7). Overall, ADGGIP achieved a favorable AUC of 0.880 (95% CI 0.685–1) in the prospective validation cohort. The prospective validation cohort performed slightly worse, possibly due to the small sample size (*n* = 31). In addition, ADGGIP also had the highest precision-recall AUC, which ranged from 0.942 to 0.963. The AUCs of the single-modality model and ClinicRad model were slightly lower than that of ADGGIP (AUC range: 0.721–0.860). For the diagnostic model based on a single diffusion technology, the DTI model (simple model) showed the highest diagnostic performance (AUC range: 0.821–0.851) in all cohorts (Supplementary Table S4). In the training cohort, each predictor contained in the rMRI, ClinicRad, CliRadDTI, and ADGGIP was used to independently predict tumor grade, with AUC values ranging from 0.502 to 0.821 (Supplementary Figure S5).

**Table 3:** Prediction performance of ADGGIP compared with other integrated prediction models

	Training cohort	Internal validation cohort	Prospective validation cohort
ADGGIP			
AUC*	0.958 (0.907–0.992)	0.942 (0.885–0.982)	0.880 (0.685–1.000)
Sensitivity	0.853 (29/34)	0.824 (28/34)	0.826 (19/23)
Specificity	0.974 (37/38)	0.947 (36/38)	0.875 (7/8)
PPV	0.967 (29/30)	0.933 (28/30)	0.950 (19/20)
NPV	0.881 (37/42)	0.857 (36/42)	0.636 (7/11)
ACC	0.917 (66/72)	0.889 (64/72)	0.839 (26/31)
CliRadDTI			
AUC*	0.913 (0.851–0.968)	0.891 (0.803–0.962)	0.924 (0.821–1.000)
Sensitivity	0.794 (27/34)	0.824 (28/34)	0.826 (19/23)
Specificity	0.921 (35/38)	0.921 (35/38)	0.875 (7/8)
PPV	0.900 (27/30)	0.903 (28/31)	0.950 (19/20)
NPV	0.833 (35/42)	0.854 (35/41)	0.636 (7/11)
ACC	0.861 (62/72)	0.875 (63/72)	0.839 (26/31)
ClinicRad			
AUC*	0.841 (0.735–0.926)	0.823 (0.727–0.923)	0.837 (0.641–0.973)
Sensitivity	0.882 (30/34)	0.853 (29/34)	0.913 (21/23)
Specificity	0.790 (30/38)	0.763 (29/38)	0.625 (5/8)
PPV	0.790 (30/38)	0.763 (29/38)	0.875 (21/24)
NPV	0.882 (30/34)	0.853 (29/34)	0.714 (5/7)
ACC	0.833 (60/72)	0.806 (58/72)	0.839 (26/31)
rMRI			
AUC*	0.860 (0.767–0.933)	0.773 (0.661–0.875)	0.870 (0.739–0.967)
Sensitivity	0.853 (29/34)	0.559 (19/34)	0.870 (20/23)
Specificity	0.737 (28/38)	0.895 (34/38)	0.625 (5/8)
PPV	0.744 (29/39)	0.826 (19/23)	0.870 (20/23)
NPV	0.849 (28/33)	0.694 (34/49)	0.625 (5/8)
ACC	0.792 (57/72)	0.736 (53/72)	0.807 (25/31)

Data in parentheses are the numerator/denominator of participants included for each parameter, unless otherwise indicated. Values correspond to the optimal threshold according to the maximum Youden index

^*^Data are the mean (95% CI)

*AUC* area under the curve, *PPV* positive predictive value, *NPV* negative predictive value, *ACC* accuracy, *ADGGIP* adult-type diffuse gliomas grade integrated prediction model (integrated model for predicting adult-type diffuse gliomas grade), *CliRadDTI* model incorporating clinical factors, radiologist interpretations and DTI data, *ClinicRad* model incorporating clinical factors and interpretations from radiologists, *rMRI* radiomics MRI

The DeLong test, integrated discrimination improvement, and net reclassification improvement were used to compare the diagnosis efficacy of the various models. Since net reclassification improvement requires artificial presetting of the cutoff point and the DeLong test is insensitive to small samples, integrated discrimination improvement was used as the gold standard when results were not matched. The results demonstrated that ADGGIP was superior to all other models (*p* < 0.05; except for the prospective validation group for the DTI model), while the DTI model was the best of the five single-modality models. The rMRI model performed worse than the DTI model (integrated discrimination improvement < 0) (Supplementary Tables S5-7).

**Fig. 4:**
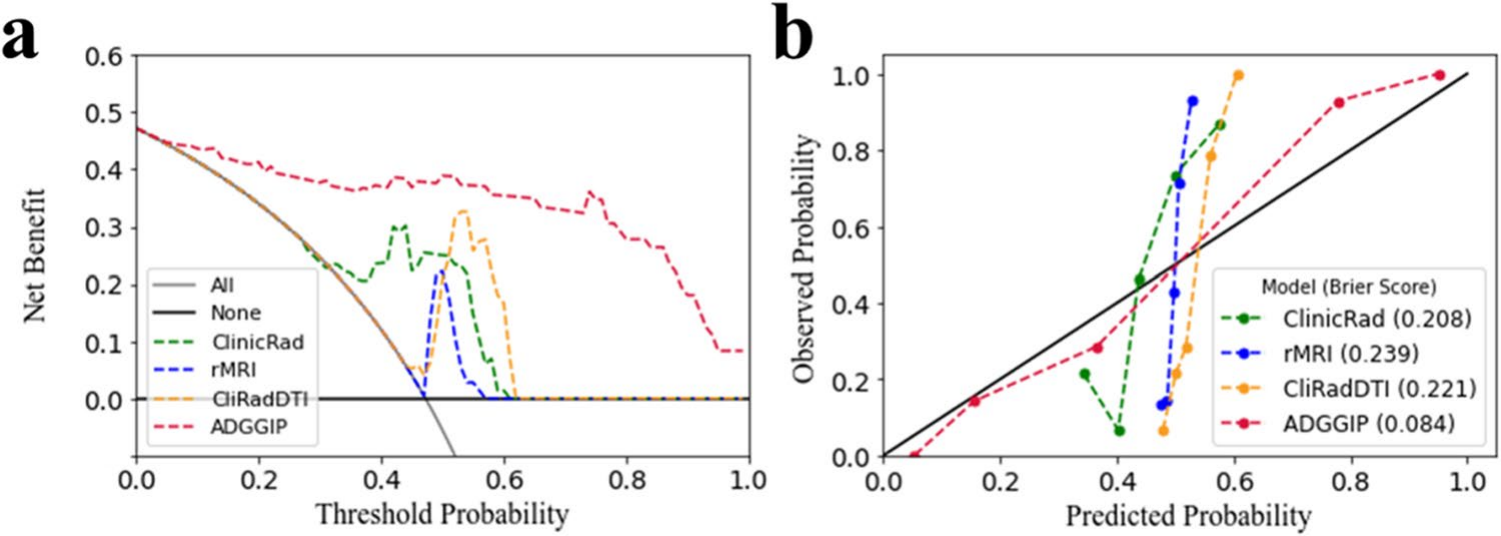
Decision curve analysis (**a**) and calibration curve (**b**) of ADGGIP versus other integrated prediction models in the training cohorts. Decision curves show that ADGGIP predicts adult-type diffuse gliomas grade better than intervention all, no intervention and other single models when the threshold is above 5%. ADGGIP predictions were closer to the real situation and had the lowest Brier score (**b**). ClinicRad, model incorporating clinical factors and interpretations from radiologists; rMRI, radiomics MRI; CliRadDTI, model incorporating clinical factors, radiologist interpretations and DTI data; ADGGIP, adult-type diffuse gliomas grade integrated prediction model

The calibration curve showed that with ADGGIP, the data generated in the training cohort had the highest consistency between the predicted value and the observed value compared with all other models (Fig. 4 and Supplementary Figure S8). In further quantitative analysis, ADGGIP had the smallest Brier score (0.084), which also verified the results of the calibration curve (Supplementary Table S8), indicating that ADGGIP composed of multimodal features was more consistent with the real situation, thus improving the prediction performance.

### Clinical value

Decision curve analysis was performed for the nine diagnostic models, and the findings are shown in Fig. 4 and Supplementary Figure S8. At threshold probabilities greater than 5%, ADGGIP provided a greater net benefit than the other eight models in predicting the grade of ADG compared with the case in which no predictive model was used (i.e., all or none).

## Discussion

In this study, we developed and validated a radiomics-based model that can assess ADG grade before treatment by combining quantitative diffusion radiomics features and clinical and radiographic morphological features. The integrated model for predicting ADG grade (ADGGIP) accurately predicted the ADG grade, had the best diagnostic efficacy and stability compared to other combination models, and showed good net benefits in decision curve analysis. And first-order traits may be a more correlated biomarker for grade prediction.

A previous study [16] identified the potential application of preoperative DWI and DTI in combination with machine learning for identifying multiple genetic traits aimed at improving the treatment of patients with glioma, particularly primary glioblastoma. They compared multiple prediction models and concluded that a comprehensive model based on radiomics and deep learning could be used to predict genetic biomarkers. However, the application of deep learning reduces the interpretability of the model, and without additional verification, there is a potential risk of overfitting. In a parallel study, several scholars carried out similar work and established four models capable of providing information correlating to different single genetic traits [13]. However, the classification criteria applied to the cohort did not fully conform to the clinical situation [17], leading to unclear clinical applicability. In this study, compared with previous studies, more accurate glioma subgroup classification methods were applied, and the model based on a single diffusion technology showed better performance (AUC range: 0.721–0.851) than the DWI (AUC range: 0.631–0.725). It is suggested that an advanced diffusion technology may be a better clinical choice. The poor correlation between ADC and histopathological features can be considered one explanation for this phenomenon [18].

The excellent predictive performance of ADGGIP may be due to the comprehensive incorporation of tumor macrostructural and microstructural features versus being limited to heterogeneous radiomics features. Gao et al [7] applied four diffusion technologies to study the main genetic information of glioma. They found that single-modality models showed similar diagnostic performances among each other, and the diagnostic models that integrated multiple diffusion modality features did not show better application value. However, they did not carry out additional clinical or higher-order feature extraction. This was improved in our study, considerably enhancing the therapeutic application potential. Even so, their integrated prediction model still showed the contribution of DTI and MAP-MRI, which was similarly reflected in our results. In addition, in our study, multiple pipelines were used to select the optimal model, versus being limited to a certain method or model, i.e., “No Free Lunch Theorem” [19]. Finally, the calibration curve and Brier scores between the models confirmed that the higher diagnostic performance of ADGGIP was more consistent with the real situation.

As advanced non-Gaussian diffusion technologies, the MAP-MRI, NODDI, and DKI should theoretically reflect the real situation of water molecular diffusion more accurately than simple Gaussian diffusion technology (DTI) and better characterize the complexity and inhomogeneity of the tissue microenvironment [20]. However, the DTI prediction model is more accurate than other single-modality prediction models. This finding means that the advantages of the integrated diffusion model may be currently only theoretical, and point-to-point studies are necessary to analyze the internal association between the predictive integrated diffusion model and pathological features. Interestingly, ADGGIP showed the highest diagnostic efficacy and stability when combined with clinical and morphologic features and was superior to the best single-modality model (DTI) combined with clinical and morphologic features. That result indicates the importance of multimodal features, and even omitting one type of feature can degrade prediction performance [21].

Some scholars believe that first-order features have more substantial associations with tumor characteristics than higher-order features [18], and energy often shows the strongest radio-tissue association. In our study, the energy of fractional anisotropy, as independent factor, was incorporated into both the DTI and rMRI models, which proved the point. Energy was distributed differently between LGG and HGG groups, which may be explained as the residual white matter fiber bundle [6, 22] and represents a more invasive pathological feature [23].

Repeatability and reproducibility are necessary for machine learning model building [10, 24]. Some scholars have found that simultaneous multi-slice technology had an impact on the extraction of diffusion features [25]. The field strength also causes some effects [26], and the higher the correlation of features to the actual situation, the smaller the effect of field strength [18]. During data collection, we did not conduct additional simultaneous multi-slice technology to reduce scanning time, but to obtain more accurate diffusion information. Nevertheless, the advanced DSI-based preprocessing technique, which involved fitting four diffusion models by scanning a single sequence, ensured the viability of clinical application. Simultaneous multislice can be used to significantly reduce the scan time in the future, although it may affect the settlement of diffusion parameters [25]. Naturally, it is likely that some centers will not be able to comply with some of the model’s requirements, such scanning for DSI. DTI might be an alternative at this stage. The diagnostic efficacy of a comprehensive predictive model based on DTI is still quite good.

Despite the promising results, our study has some limitations. First, the comprehensive diagnostic model was based on data from a single center. However, comprehensive and effective statistical analysis methods ensure the accuracy and stability of the model. Second, manual delineation of tumor regions of interest is the current gold standard, but it takes considerable time and effort. In the future, fully automated tumor segmentation based on machine learning will be incorporated into practical applications, such as neural networks. Third, there were not enough prognostic data to support the usability of the model, which needs to be further explored and validated in future studies.

In conclusion, we present ADGGIP, a noninvasive and accurate radiomic model that combines radiomic, clinical, and imaging morphological features to facilitate the preoperative assessment of WHO grade in patients with adult-type diffuse gliomas. The performance and stability of ADGGIP highlight the potential of advanced diffusion models for precision therapy in patients with adult-type diffuse gliomas.

### Supplementary Information

Below is the link to the electronic supplementary material.

Supplementary file1 (PDF 3.40 MB)

## Abbreviations

ADG: Adult-type diffuse gliomas
ADGGIP: Adult-type diffuse gliomas grade integrated prediction model
AUC: Area under the curve
ClinicRad: Model incorporating clinical factors and interpretations from radiologists
CliRadDTI: Model incorporating clinical factors, radiologist interpretations and DTI data
CNS: Central nervous system
DKI: Diffusion kurtosis imaging
DSI: Diffusion spectrum magnetic resonance imaging
DTI: Diffusion tensor imaging
HGG: High-grade glioma
LGG: Low-grade glioma
MAP-MRI: Mean apparent propagation diffusion-MRI
NODDI: Neurite orientation dispersion and density imaging
rMRI: Radiomics MRI
WHO: World Health Organization

## Declarations

### Guarantor

The scientific guarantor of this publication is Yang Gao.

### Statistics and biometry

Yang Song kindly provided statistical advice for this manuscript.

### Study subjects or cohorts overlap

Some study subjects or cohorts have been previously reported in “Wang P, Weng L, Xie S, et al Primary application of mean apparent propagator-MRI diffusion model in the grading of diffuse glioma. Eur J Radiol 2021;138:109622,” “Xie SH, Lang R, Li B, et al. Evaluation of diffuse glioma grade and proliferation activity by different diffusion-weighted-imaging models including diffusion kurtosis imaging (DKI) and mean apparent propagator (MAP) MRI. Neuroradiology. 2023;65(1):55-64, ” “He J, Ren J, Niu G, et al Multiparametric MR radiomics in brain glioma: models comparation to predict biomarker status. BMC Med Imaging. 2022;22(1):137. Published 2022 Aug 5,” and “Wang P, He J, Ma X, et al. Applying MAP-MRI to Identify the WHO Grade and Main Genetic Features of Adult-type Diffuse Gliomas: A Comparison of Three Diffusion-weighted MRI Models. Acad Radiol. 2023;30(7):1238-1246.”

### Methodology

• prospective

• diagnostic or prognostic study

• performed at one institution
