# Supplementary material for: Model incorporating multiple diffusion MRI features: development and validation of a radiomics-based model to predict adult-type diffuse gliomas grade
Source: Eur Radiol. 2023 Jul 13;33(12):8809–20. doi: 10.1007/s00330-023-09861-0 (PMC10667393; doi:10.1007/s00330-023-09861-0)
Supplement: Supplementary file 1 — Supplementary file1 (PDF 3.40 MB) [file 330_2023_9861_MOESM1_ESM.pdf]

Model incorporating multiple diffusion MRI features: development and validation of a radiomics-based model to predict adult-type diffuse gliomas grade

Electronic Supplementary Material

Research in Context

Evidence before this study

We searched the PubMed database from January 1, 2020 to August 10, 2022, for articles on artificial intelligence-based methods to predict the pathological features of gliomas before treatment, including histological grade and major genetic features, mainly by magnetic resonance scanning. We used the search terms "artificial intelligence" or "machine learning" or "deep learning" or "radiomics" or "radiogenomic", "MRI", "glioma" or "glioblastoma" without language restrictions. We identified 92 original studies that applied machine learning to predict histological grade or associated genetic features, highlighting the potential and advantages of machine learning methods for the preoperative prediction of pathological features in gliomas, with one-third of these studies reporting the value of using machine learning methods with MRI for glioma grading.

Despite encouraging preliminary results, the clinical applicability of these methods remains unclear due to the retrospective nature of these studies, the lack of validation, and flawed analytical methods. In addition, the lack of publicly available algorithm codes undermines the transparency and reproducibility of these prediction systems. Studies typically focus on only one aspect of a histological trait or genetic trait. In 60% of all studies, only conventional MRI was applied for exploratory analysis and did not involve other functional imaging or other imaging data. However, the application of multimodal data actually facilitated the development of comprehensive diagnostic models, such as diffusion models or perfusion models. There are only 4 original studies that have involved the analysis of 2 diffusion models simultaneously. To our knowledge, no study has prospectively validated the potential benefits of combining radiomic, clinical features, and imaging features using multiple diffusion models to improve the performance of artificial intelligence models to predict adult-type diffuse gliomas grade.

Added value of this study

In this prospective study, we developed and validated an integrated diagnostic model (Adult-type Diffuse Gliomas Grade Integrated Prediction model; ADGGIP) to predict the WHO grade of adult-type diffuse

gliomas before treatment. ADGGIP was constructed by machine learning, extracting key factors from multiple diffusion modalities (diffusion tensor imaging [DTI], diffusion kurtosis imaging [DKI], neurite orientation dispersion and density imaging [NODDI] and mean apparent propagator [MAP]-MRI), clinical features, and imaging features. ADGGIP was able to effectively differentiate the pathological grades of adult-type diffuse gliomas and significantly outperformed prediction models constructed from a single factor. The predictive performance of ADGGIP was validated with both internal and time-based external datasets. Time-based external datasets were applied for validation, highlighting the robustness of ADGGIP.

Implications of all the available evidence

ADGGIP can help effectively predict the pretreatment grade of adult-type diffuse gliomas. The 2021 edition of the WHO Classification of Tumors of the Central Nervous System facilitates the development of personalized treatment regimens, especially in a period of transition where targeted and immunotherapy modalities have become prominent and important. In future clinical practice, the use of ADGGIP could promote treatment options for patients considered suitable for drug therapy and expand the survival benefits of surgical treatment for patients with highly malignant tumors.

List of Appendixes

Part I. Supplementary Information

I. Magnetic Resonance Scanning

II. Pathological Diagnosis

III. Preprocessing

IV. Region of Interest Segmentation

V. Radiomics Feature Extraction

VI. Imaging Feature Extraction Criteria

VII. Balancing Data and Normalization/Standardization

VIII. Feature Dimensionality Reduction and Selection

IX. Modeling and Validation Strategy

X. Sample Size and Power Calculations

Part II. Supplementary Figures

Figure S1. Four diffusion models and their parameter diagrams

Figure S2. Heatmaps of feature correlation

Figure S3. Heatmaps of feature contributions

Figure S4. Flow chart of nine prediction models

Figure S5. Importance (a) of multimodal variables and performance (b) of the machine learning models that ADGGIP was based on

Figure S6. Two radiomics signatures obtained from ADGGIP

Figure S7. Prediction profiles of ADGGIP

Figure S8. Decision curve analysis (a) and calibration curves (b) of single-modality predictionmodels in the training cohorts

Part III. Supplementary Tables

Table S1. Anatomical and diffusion MRI parameters

Table S2. Radiomic features extracted by FeAture Explorer

Table S3. Adult-type diffuse gliomas classification proportions among cohorts

Table S4. Prediction performance of single-modality models

Table S5. Delong test for ROC curve improvements in multiple cohorts

Table S6. IDI test for prediction improvements in multiple cohorts

Table S7. NRI test for prediction improvements in multiple cohorts

Table S8. Brier score for ADGGIP in achieving prediction improvements compared with other models in the training cohort

Supplementary Information

I. Magnetic Resonance Scanning

MRI scans were performed when participants entered the cohort. All study participants underwent preoperative MRI using a 3T scanner (MAGNETOM Skyra; Siemens Healthcare, Erlangen, Germany) equipped with a 32-channel head/neck coil. Scans were performed over the same time period to maximize the reproducibility of the radiographic features [1].

The conventional MRI (cMRI) sequences included axial T1-weighted, axial T2-weighted, axial T2-weighted FLAIR, and 3D contrast-enhanced T1-weighted images after the intravenous administration of 0.1 mmol/kg gadobutrol (Gadovist, Bayer AG, Berlin, Germany). Diffusion imaging sequences included axial diffusion-weighted imaging (DWI) and diffusion spectrum magnetic resonance imaging. Detailed information on the parameters is provided in Supplementary Table S1.

II. Pathological Diagnosis

All tissue samples were prepared as paraffin blocks and analyzed at our institution's pathology department using the latest methodology consistent with the 2021 World Health Organization (WHO) guidelines on histopathology and immunohistochemistry [2]. Genetic characteristics were detected by a one-step method (multiplex PCR amplification combined with next-generation sequencing [NGS]) [3, 4]. NGS has the characteristics of being unbiased and having wide coverage, high sensitivity and high speed. The pathologist (Lixin Weng) who analyzed the images had 23 years of work experience and was blinded to the clinical information and imaging results. The subjectivity of pathologists may result in misclassification of histological grades, but the 2021 WHO Classification of Tumors of the Central Nervous System makes the actual grading more dependent on

molecular typing, effectively reducing the incidence of misclassification.

NGS steps: A Maxwell® RSC FFPE Plus DNA Purification Kit (Promega AX4920) was used to extract DNA from formalin-fixed paraffin-embedded (FFPE) tissues. DNA was quantified using the Qubit dsDNA HS Assay Kit (Thermo Fisher Scientific; Q32851). A six-item glioma detection kit (Genetron Health Technology, Co., Ltd.) was used to build the DNA library, Agencourt AMPure XP (Beckman Coulter; A63880) magnetic beads were used to purify the library, and an Agilent TapeStation 2200 quantitative kit and Qubit dsDNA HS Assay Kit (Thermo Fisher Scientific; Q32851)were used for fragment quality control and quantification.

Sequencing was performed using the Ion Torrent next-generation high-throughput sequencing platform. Template preparation and the on-machine sequencing process were performed according to the instructions provided with the general sequencing reaction kit (Genetron Health Technology, Co., Ltd.). The fully automated sample addition system GENETRON Chef produced by Genetron Health Technology, Co., Ltd. was used for template preparation, and the gene sequencer GENETRON S5 produced by Genetron Health Technology, Co., Ltd. was used to sequence the samples. After sequencing was completed, built-in software BaseCaller (GENETRON S5: v5.8.10) provided with the sequencer was used for base identification and base information statistics, and the built-in software TMAP (GENETRON S5: v5.8.10) was used to complete the sequencing results and align the resulting sequences to the human reference genome hg19 (GRCh37). Next, the built-in plug-in of the sequencervariantCaller (GENETRON S5: v5.8.0.19) and the ghall plug-in developed by Genetron Health Technology, Co., Ltd. were used to perform mutation analysis and annotate theoffline data as well as generate automated reports.

III. Preprocessing

Diffusion parameters were calculated using NeuDiLab, a software developed in-house with Python based on the open-source tool DIPY (Diffusion Imaging in Python, https://dipy.org) [5]. The software is equipped with FSL-based brain extraction, eddy current and head motion correction, and smoothing functions [6]. Final quantitative parametric maps for 25 features from the 4 diffusion models and B0 maps were obtained. Three advanced models (diffusional kurtosis imaging [DKI], neurite orientation dispersion and density imaging [NODDI] and mean apparent propagation diffusion magnetic resonance imaging [MAP-MRI]) and 1 simple model (diffusion tensor imaging [DTI]) were included (Supplementary Figure S1) [7-10]. The cMRI scans were bias corrected using the N4ITK MRT Bias correction module in 3D-Slicer [11]. Diffusion parameter maps were not bias corrected or intensity normalized because diffusion parameters are a quantitative measure [12] and may maintain a relatively consistent trend without bias correction [13]. Coregistration of cMRI and diffusion parameter maps was performed using the General Registration module (based on ANTs) in 3D-Slicer.

IV. Region of Interest Segmentation

Semiautomatic selection of regions of interest (ROIs) was performed by two radiologists (Peng Wang and Zhiyue Hao, with 3 and 2 years of neuroimaging experience, respectively) using 3D Slicer under the supervision of another senior physician (Yang Gao, with 27 years of neuroimaging experience). The three radiologists were aware of the tumor diagnosis but were blinded to the clinical and pathological details. The ROIs were outlined on B0 maps with reference to the cMRI. The area of tumor accumulation was selected as the ROI. The areas of the solid tumor and peritumoral edema were usually radiologically described as the area

surrounded by abnormal/high signals on T2-FLAIR. Areas located around edema that were suspected to be invaded, such as those with slightly high signal or other abnormal signal patterns found on T2WI, were usually also included in the ROI. Finally, the mask generated by the 3D-ROIs was assigned to each parameter map for feature extraction.

To ensure the repeatability of manual segmentation and feature extraction, the above steps were repeated after two weeks. For the extracted radiomics features, intraclass correlation coefficients (ICCs) were used to assess intra- and interobserver agreement. In this study, the ICC values for the radiological features extracted all reached 0.60 or higher. We did not set a higher threshold because poor reproducibility of radiomic features does not necessarily translate into poor disease discrimination. That is, the specific values of the features may have changed significantly, but their relative order may not have changed [14].

V. Radiomics Feature Extraction

FeAture Explorer (FAE v0.5.2, https://github.com/salan668/FAE) software was used to extract radiomics parameters [15]. Feature extraction was partially performed by PyRadiomics (version 3.0) [16]. The mathematical definitions of the imaging features can be found on the PyRadiomics website (https://pyradiomics.readthedocs.io/en/latest/radiomics.html). In total, there were 107 characteristic indicators of the original images (Supplementary Table S2), including first-order features [n=18], shape-based features [n=14], gray level cooccurrence matrix (GLCM) [n=24], gray level dependence matrix (GLDM) [n=14]; gray level run length matrix (GLRLM) [n=16], gray level size zone matrix (GLSZM) [n=16], and neighboring gray tone difference matrix (NGTDM) [n=5]. Twenty-five diffusion parameters and B0 maps were used for feature extraction. In total, 2782 radiomics

features (all features were continuous data) were obtained within the 3D-ROI. cMRI has not been used for feature extraction because registration may affect its signal, which is not conducive to the repeatability and reproducibility of the study [13].

VI. Imaging Feature Extraction Criteria

Two radiologists (Shenghui Xie and Jinlong He, with 11 and 12 years of neuroimaging experience, respectively) analyzed all cMRI and DWI images independently to assess the morphologic information of each participant with adult-type diffuse gliomas. The two radiologists were aware of the tumor diagnosis but were blinded to the clinical and pathological details. In addition, judgments were broken down into independent tasks, evaluating one well-defined trait at a time while blinded to the other evaluations. When there was disagreement, a senior physician (Qiong Wu, with 15 years of neuroimaging experience) was consulted, and a final decision was then made. This work was completed within two weeks.

Both the solid tumor and intratumor edema were evaluated using the imaging features. The solid tumor components evaluated included the presence of necrosis, cystic regions, calcification, and hemorrhage, as well as the tumor's enhancement pattern, location, and side and clarity of the solid tumor boundary [17]. The minimum length (from the solid tumor to the adjacent white matter) was evaluated in the peritumoral edema region. The classification standards applied were as follows:

- Necrosis: An area of the tumor body, either patchy or irregular in shape, with a signal intensity similar to but still different from that of cerebrospinal fluid (CSF), with an enhanced edge around the necrosis area.
- Cyst or cysts: Unlike necrosis, the signal was equivalent to the CSF signal, and marginal enhancement was not significant or absent.
- Calcification and hemorrhage: Determined on the basis of T1-weighted imaging,

T2-weighted imaging, computed tomography (CT) and susceptibility weighted imaging (SWI) together (additional CT or SWI scans were performed in approximately 65% of the study subjects, a condition that would increase the uncertainty of the results).

- Tumor enhancement patterns: According to the shape and degree of enhancement, the contrast agent patterns were divided into patchy (enhancement degree: slight), annular or central (enhancement degree: obvious), and no enhancement.
- Location and side of tumor: The determination was made according to the central location of the tumor. In the case of multiple lesions, the center of the lesion with the largest volume was analyzed.
- Boundary clarity: On T2-weighted imaging, if more than 50% of the tumor margin area could not be effectively distinguished, the boundary was judged to be blurred.

Edema extent: On T2-weighted imaging, the edema extent was determined in the largest aspect of the tumor entity. When the solid boundary of the tumor was clear (e.g., the tumor showed annular enhancement at the edge), the area of T2 hyperintensity around the tumor was considered edema. When the solid tumor and edema could not be distinguished, T2 hyperintense areas that were closer to adjacent brain tissue and showed hyperintensity on apparent diffusion coefficient (ADC) maps (areas of cytotoxic edema may be more characterized by unrestricted water molecules than solid tumors) were defined as edema. Finally, the minimum extent of edema (perpendicular to the edge of the tumor) was recorded, and whether it exceeded 1.5 cm was determined. The minimum extent was chosen because it was likely to be more representative of the invasion of tumor cells, and 1.5 cm was derived from our clinical work experience.

VII. Balancing Data and Normalization/Standardization

FAE provides random upsampling, random downsampling, and SMOTE methods to achieve data balancing (the data balancing operation is performed only for the training set, the test set data are not processed, and the original training set data are stored). We chose the random upsampling method to balance the training set.

Because the intensity values of diffusion parameters are widely distributed, three feature normalization/standardization methods were considered, including mean normalization, min–max normalization, and Z score normalization. The normalized values (a) of the image intensities (x) were calculated as follows:

$$\text{Mean normalization:a} = \frac{X - X_{Mean}}{X_{Max} - X_{Min}},$$

$$\text{Min-Max normalization:a} = \frac{X - X_{Min}}{X_{Max} - X_{Min}},$$

$$\text{Z score normalization:a} = \frac{X - X_{Mean}}{X_{Std}}.$$

VIII. Feature Dimensionality Reduction and Selection

Because the number of features is much larger than the number of samples in some cases, the data were processed by reducing the number of features to subsequently perform efficient feature selection and observing model building. Both principal component analysis (PCA) and Pearson correlation coefficients (PCCs) were used to reduce the number of features (Supplementary Figures S2 and S3).

- PCA can map potentially correlated high-dimensional features to linearly uncorrelated low-dimensional features and the mapped low-dimensional data. Each feature of the mapped low-dimensional data is linearly independent.
- The PCCs are calculated two by two, and all features are traversed. When the

coefficient was greater than a certain threshold (0.85 in this study), one of them was removed randomly, avoiding high similarity with reduced-dimensional features.

After feature dimensionality reduction, four feature selection methods were selected, including analysis of variance (ANOVA), recursive feature elimination (RFE), Kruskal–Wallis (KW) and relief.

- ANOVA: Through statistical analysis of multiple variables, the weight of the F value was calculated. After sorting the values from the largest to the smallest, the most relevant features for the model were determined.
- RFE: The main idea of recursive feature elimination is to repeatedly build a model (such as a support vector machine [SVM] or regression model), pick the best (or worst) features (which can be selected based on coefficients), set the selected features aside, and then repeat the process on the remaining features until all the features have been traversed. The order in which features are eliminated in this process is the ranking of features.
- KW: Kruskal–Wallis is a nonparametric test of three or more sets of data. It is used to test the original hypothesis for consistency of the overall functional distribution and its alternative hypothesis, the hypothesis that there is a difference between at least two samples.
- Relief: The correlation between features and categories is based on the ability of features to distinguish close samples.

IX. Modeling and Validation Strategy

We analyzed ten machine learning classifiers to determine the best model. Based on the clinical interpretability of the output, we classified these classifiers into two categories: linear (logistic regression [LR], logistic regression via least absolute

shrinkage and selection operator [LR-Lasso], linear discriminant analysis [LDA], and support vector machine [SVM]) and nonlinear (autoencoder [AE], decision trees [DT], random forest [RF], ada-boost [Ab], Gaussian process [GP] and native Bayes [NB]). Explanations of each model can be found on the official website of scikit-learn (https://scikit-learn.org/stable/).

Internal and external validations were carried out. Internal validation was performed using leave-one-out cross-validation. Each time, only one sample was left for testing, and the other samples were used for construction. For K samples, the building and learning process needs to be repeated K times and tested K times. This process achieves the highest sample utilization rate, and the result is the closest to the expected value from training the whole test set. The model was then reconstructed if the internal validation set did not yield satisfactory results after the number of predictors was increased or decreased. That is, the number of features selection procedure was performed inside the cross-validation. Prospective validation was performed using a time-independent test set.

The diagnostic performance of the model was evaluated with ROC curves and Brier scores (calculated as the mean square error of the probability prediction relative to the test sample, ranging from 0 to 1, with higher scores indicating worse prediction results and worse calibration) in the training set and the internal validation set, while the performance in the prospective validation set was unknown. The above work was repeated until all the pipelines were run. Finally, the established model was tested in the prospective validation set. The selection of the comprehensive model weighed the diagnostic ability and stability of the models.

X. Sample Size and Power Calculations

The study consecutively enrolled approximately 70 subjects. Regarding AUC (H1), 70 subjects were needed for the study to detect an AUC of 0.80 for ADGGIP at ~85% power and alpha = 0.05 (two-sided). The sample size calculation was based on the following assumptions: 1) the AUC in the null hypothesis was 0.60, and 2) the positive cases accounted for 66% of the population.

The sample size and power calculations were performed in PASS 2021 software.

References

1. Hoebel KV, Patel JB, Beers AL, et al (2020) Radiomics Repeatability Pitfalls in a Scan-Rescan MRI Study of Glioblastoma. Radiol Artif Intell. DOI: 10.1148/ryai.2020190199
2. Louis DN, Perry A, Wesseling P, et al (2021) The 2021 WHO Classification of Tumors of the Central Nervous System: a summary. Neuro Oncol. DOI: 10.1093/neuonc/noab106
3. Higa N, Akahane T, Yokoyama S, et al (2020) A tailored next-generation sequencing panel identified distinct subtypes of wildtype IDH and TERT promoter glioblastomas. Cancer Sci. DOI: 10.1111/cas.14597
4. Zacher A, Kaulich K, Stepanow S, et al (2017) Molecular diagnostics of gliomas using next generation sequencing of a glioma-tailored gene panel. Brain Pathol. DOI: 10.1111/bpa.12367
5. Garyfallidis E, Brett M, Amirbekian B, et al (2014) Dipy, a library for the analysis of diffusion MRI data. Front Neuroinform. DOI: 10.3389/fninf.2014.00008
6. Jenkinson M, Beckmann CF, Behrens TE, Woolrich MW, Smith SM (2014) FSL. Neuroimage. DOI: 10.1016/j.neuroimage.2011.09.015
7. Alexander AL, Lee JE, Lazar M, Field AS (2007) Diffusion tensor imaging of the brain. Neurotherapeutics. DOI: 10.1016/j.nurt.2007.05.011
8. Jensen JH, Helpern JA, Ramani A, et al (2005) Diffusional kurtosis imaging: the quantification of non-gaussian water diffusion by means of magnetic resonance imaging. Magn Reson Med. DOI: 10.1002/mrm.20508
9. Zhang H, Schneider T, Wheeler-Kingshott CA, et al (2012) NODDI: practical in vivo neurite orientation dispersion and density imaging of the human brain. Neuroimage. DOI: 10.1016/j.neuroimage.2012.03.072
10. Özarslan E, Koay CG, Shepherd TM, et al (2013) Mean apparent propagator (MAP) MRI: a novel diffusion imaging method for mapping tissue microstructure. Neuroimage. DOI: 10.1016/j.neuroimage.2013.04.016
11. Fedorov A, Beichel R, Kalpathy-Cramer J, et al (2012) 3D Slicer as an image computing platform for the Quantitative Imaging Network. Magn Reson Imaging. DOI: 10.1016/j.mri.2012.05.001
12. Bobholz SA, Lowman AK, Barrington A, et al (2020) Radiomic Features of Multiparametric MRI Present Stable Associations With Analogous Histological Features in Patients With Brain Cancer. Tomography. DOI: 10.18383/j.tom.2019.00029
13. Shiri I, Hajianfar G, Sohrabi A, et al (2020) Repeatability of radiomic features in magnetic resonance imaging of glioblastoma: Test- retest and image registration analyses. Med Phys. DOI: 10.1002/mp.14368

14. Lv W, Yuan Q, Wang Q, et al (2018) Robustness versus disease differentiation when varying parameter settings in radiomics features: application to nasopharyngeal PET/CT. Eur Radiol. DOI: 10.1007/s00330-018-5343-0
15. Song Y, Zhang J, Zhang YD, et al (2020) FeAture Explorer (FAE): A tool for developing and comparing radiomics models. PLoS One. DOI: 10.1371/journal.pone.0237587
16. van Griethuysen JJM, Fedorov A, Parmar C, et al (2017) Computational Radiomics System to Decode the Radiographic Phenotype. Cancer Res. DOI: 10.1158/0008-5472.CAN-17-0339
17. Maynard J, Okuchi S, Wastling S, et al (2020) World Health Organization Grade II/III Glioma Molecular Status: Prediction by MRIMorphologic Features and Apparent Diffusion Coefficient. Radiology. DOI: 10.1148/radiol.2020191832

Supplementary Figures

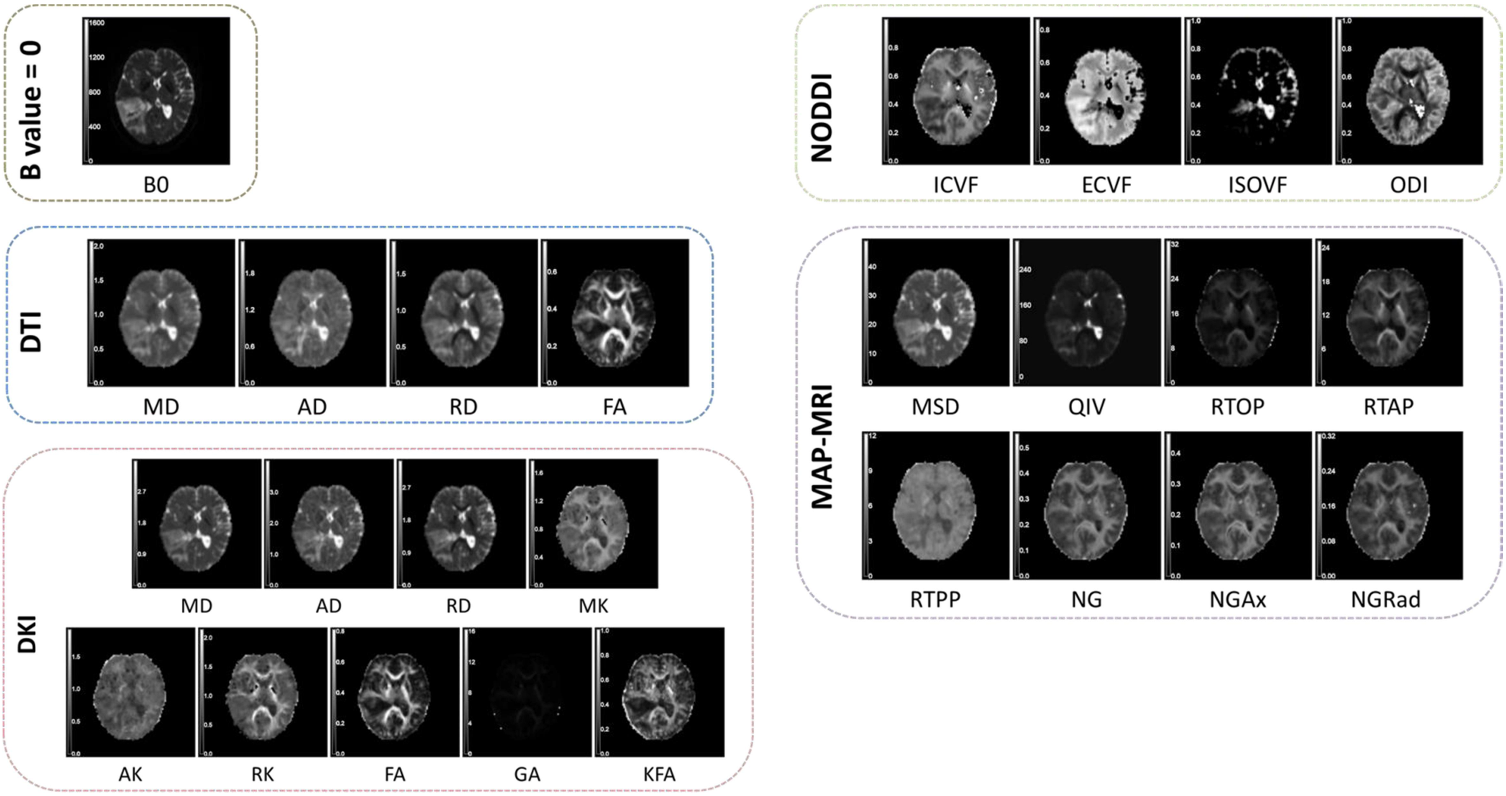

Figure S1. Four diffusion models and their parameter diagrams

B0 = diffusion b0 parameter diagram, DTI = diffusion tensor imaging, DKI = diffusion kurtosis imaging, NODDI = neurite orientation dispersion and density imaging, MAP-MRI = mean apparent propagation diffusion magnetic resonance imaging. MD = mean diffusivity, AD = axial diffusivity, RD = radial diffusivity, FA = fractional anisotropy, MK = mean kurtosis, AK = axial kurtosis, RK = radial kurtosis, GA = generalized fractional anisotropy, KFA = kurtosis fractional anisotropy, ICVF = intracellular volume fraction, ECVF = extracellular volume fraction, ISOVF = isotropic or free water volume fraction, ODI = orientation dispersion index, MSD = mean squared displacement, QIV = q-space inverse variance, RTOP = return to the origin probability, RTAP = return to the axis probability, RTPP = return to the plane probability, NG = non-Gaussianity, NGAx = non-Gaussianity axial, NGRad = non-Gaussianity vertical.

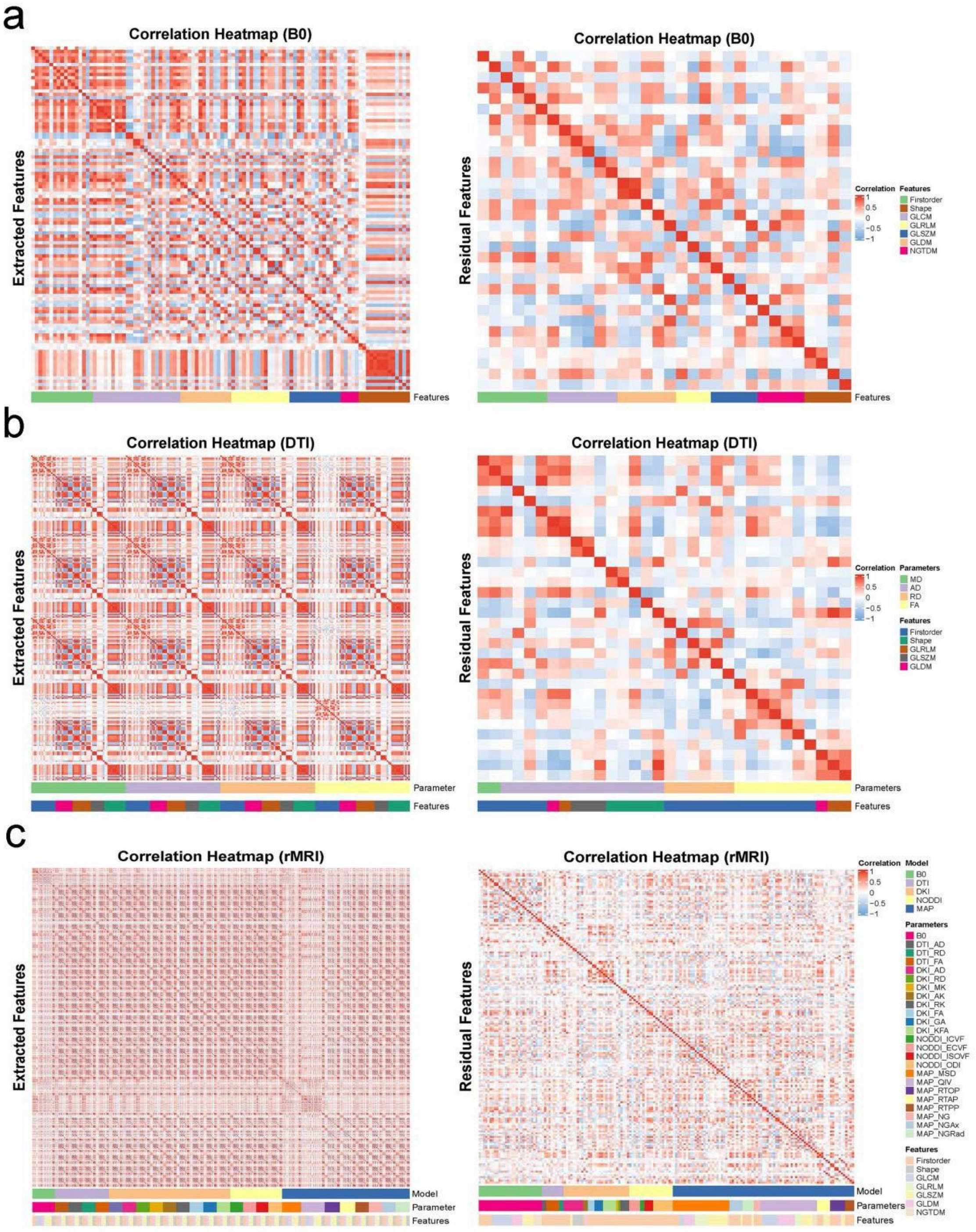

Figure S2. Heatmaps of feature correlation

Mutual correlations among individual features of B0 (a), DTI (b) and rMRI (c) were calculated by Pearson coefficients. B0 had 32 residual features, DTI had 32, and rMRI had 172. Redundant correlation features were significantly diminished when the Pearson correlation coefficients was set at 0.85.
B0 = diffusion b0 parameter diagram, DTI = diffusion tensor imaging, rMRI = radiomics MRI, GLCM = gray level cooccurrence matrix, GLRLM = gray level run length matrix, GLSZM = gray level size zone matrix, GLDM = gray level dependence matrix, NGTDM = neighborhood gray-tone difference matrix.

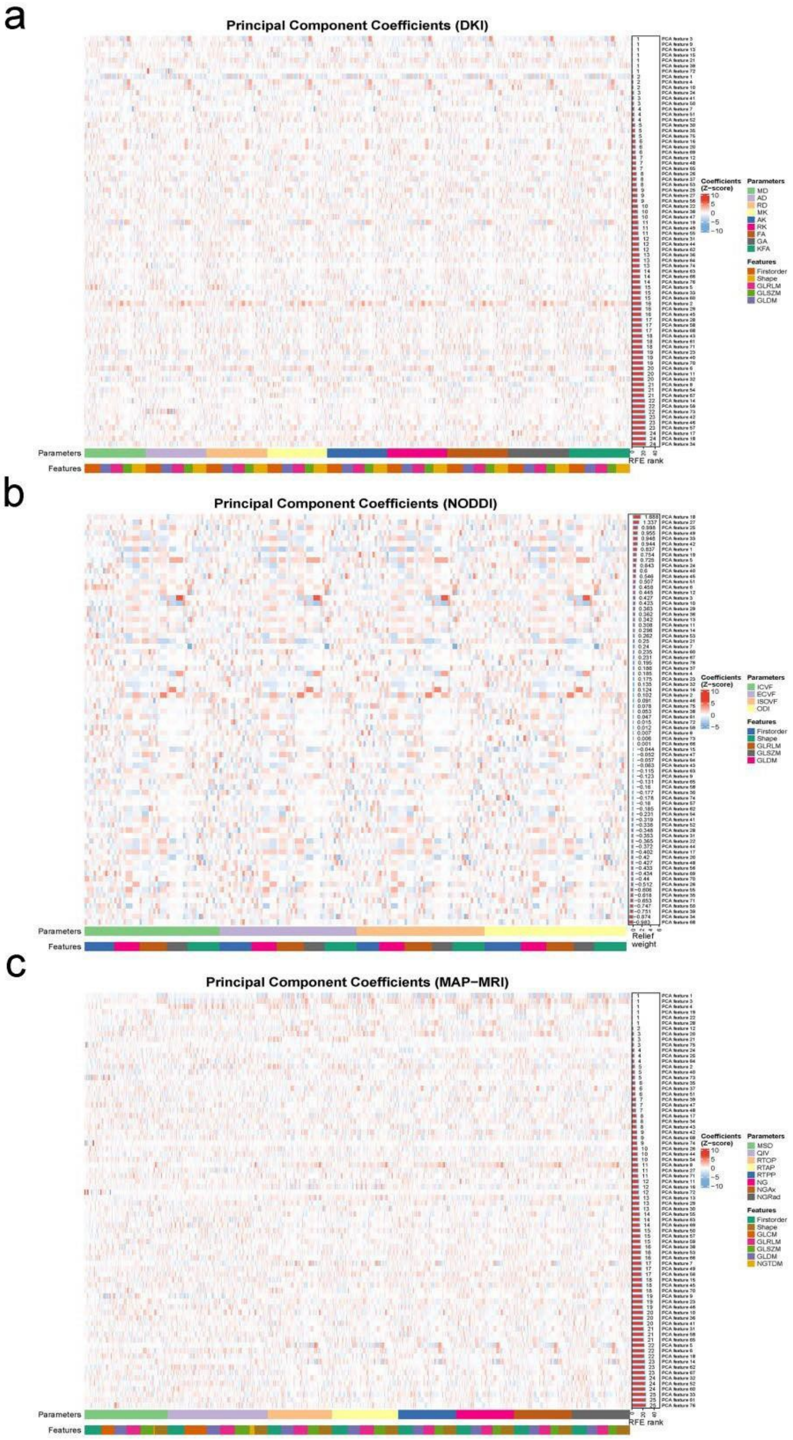

Figure S3. Heatmaps of feature contributions

Principal component coefficients among individual features and principal components for DKI (a), NODDI (b) and MAP-MRI (c) were calculated by principal component analysis. Overfitting was prevented by the use of principal component analysis, which decreased the amount of features.
DKI = diffusion kurtosis imaging, NODDI = neurite orientation dispersion and density imaging, MAP-MRI = mean apparent propagation diffusion magnetic resonance imaging, RFE = recursive feature elimination, GLCM = gray level cooccurrence matrix, GLRLM = gray level run length matrix, GLSZM = gray level size zone matrix, GLDM = gray level dependence matrix, NGTDM = neighborhood gray-tone difference matrix.

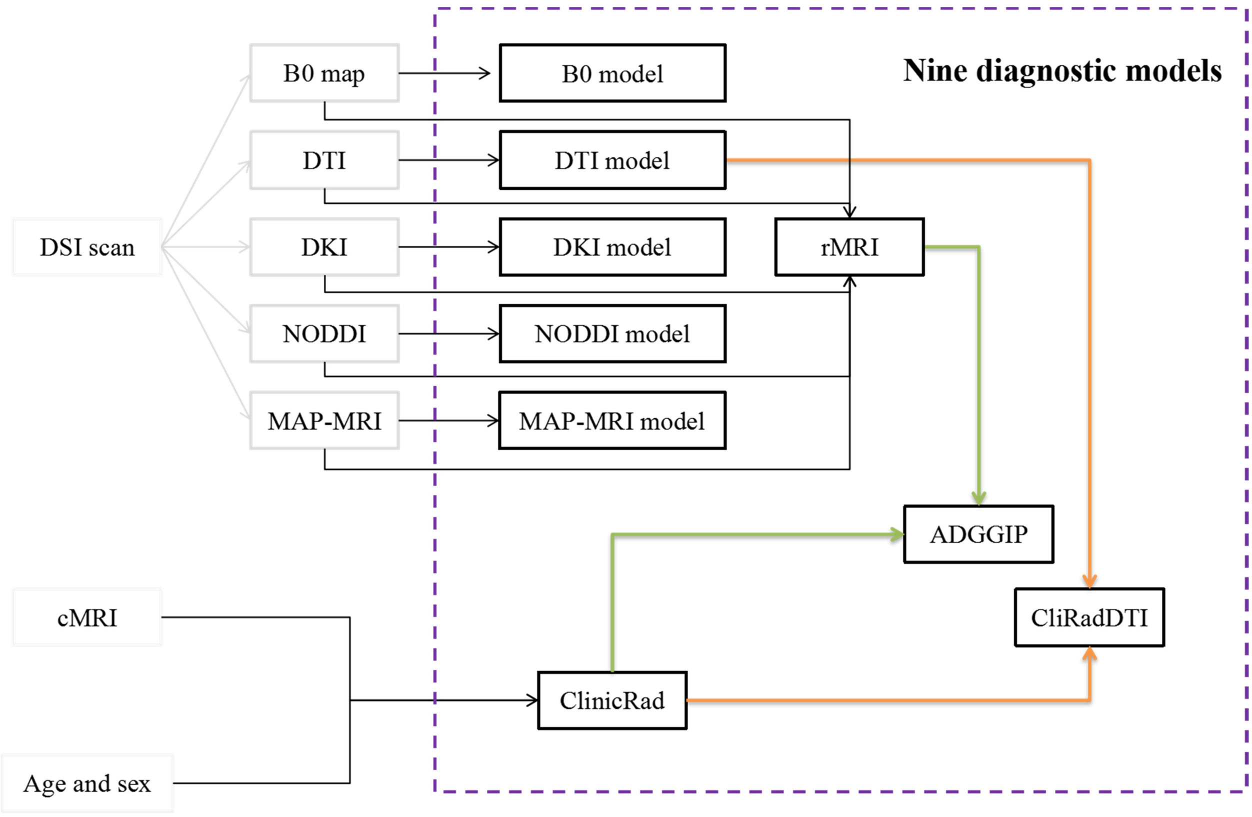

Figure S4. Flow chart of nine prediction models

Note: The DTI model was the single-modality model with the highest diagnostic performance in the prospective validation cohort, and rMRI was the more theoretically relevant prediction model incorporating multiple diffusion features.

B0 = diffusion b0 parameter diagram, DTI = diffusion tensor imaging, DKI = diffusion kurtosis imaging, NODDI = neurite orientation dispersion and density imaging, MAP-MRI = mean apparent propagation diffusion magnetic resonance imaging. cMRI = conventional MRI, ClinicRad = model incorporating clinical factors and interpretations from radiologists, rMRI = radiomics MRI, CliRadDTI = model incorporating clinical factors, radiologist interpretations and DTI data, ADGGIP = Adult-type Diffuse Gliomas Grade Integrated Prediction model

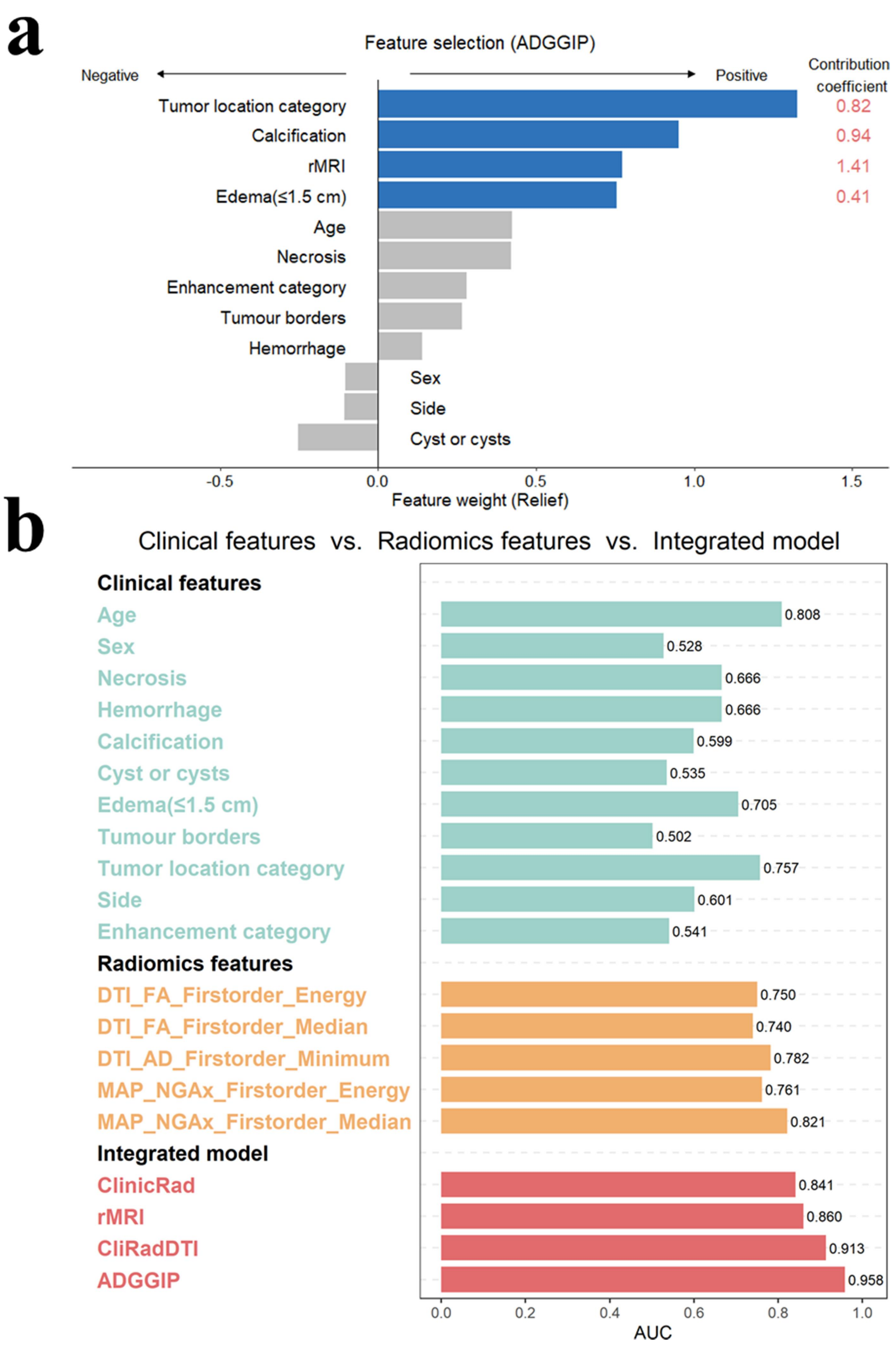

Figure S5. Importance (a) of multimodal variables and performance (b) of the machine learning models that ADGGIP was based on

The log odds ratios for ADGGIP were as follows: L= (0.82 × tumor location category) + (0.94 × calcification) + (1.41 × rMRI) + (0.41 × edema(≤1.5 cm)) + 0.17. The probability of HGG was calculated for ADGGIP by using the equation $1/(1 + e^{-L})$, where L is the relevant log odds ratio. DTI = diffusion tensor imaging, FA = fractional anisotropy, AD = axial diffusivity, MAP = mean apparent propagation diffusion, ClinicRad = model incorporating clinical factors and interpretations from radiologists, rMRI = radiomics MRI, CliRadDTI = model incorporating clinical factors, radiologist interpretations and DTI data, ADGGIP = Adult-type Diffuse Gliomas Grade Integrated Prediction model.

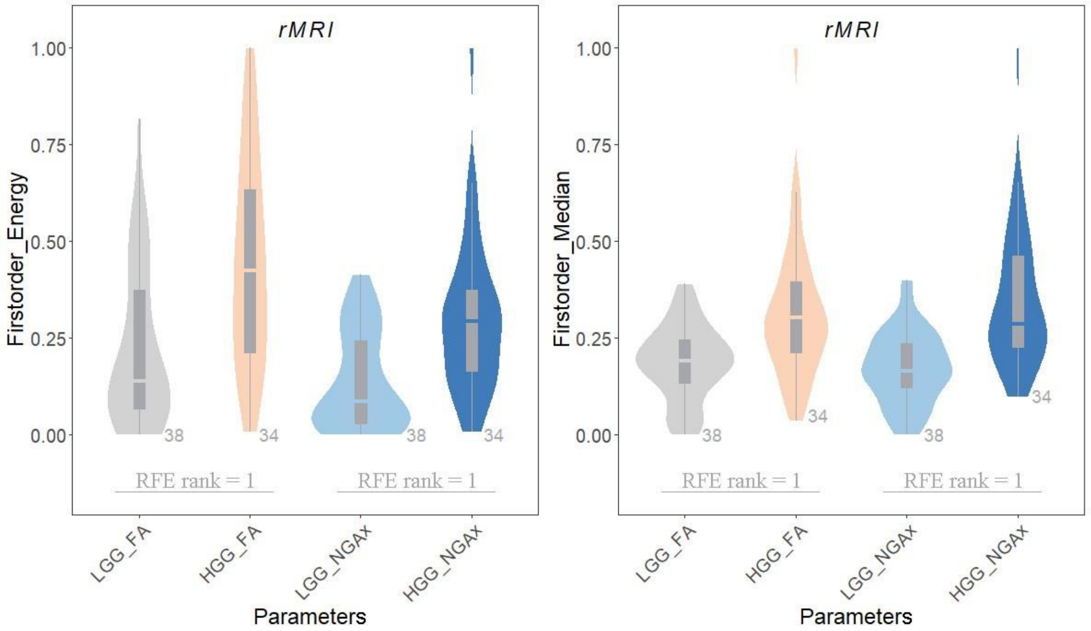

Figure S6. Two radiomics signatures obtained from ADGGIP

LGG = low-grade glioma, HGG = high-grade glioma. FA = fractional anisotropy, NGAx =non-Gaussianity axial. RFE = recursive feature elimination.

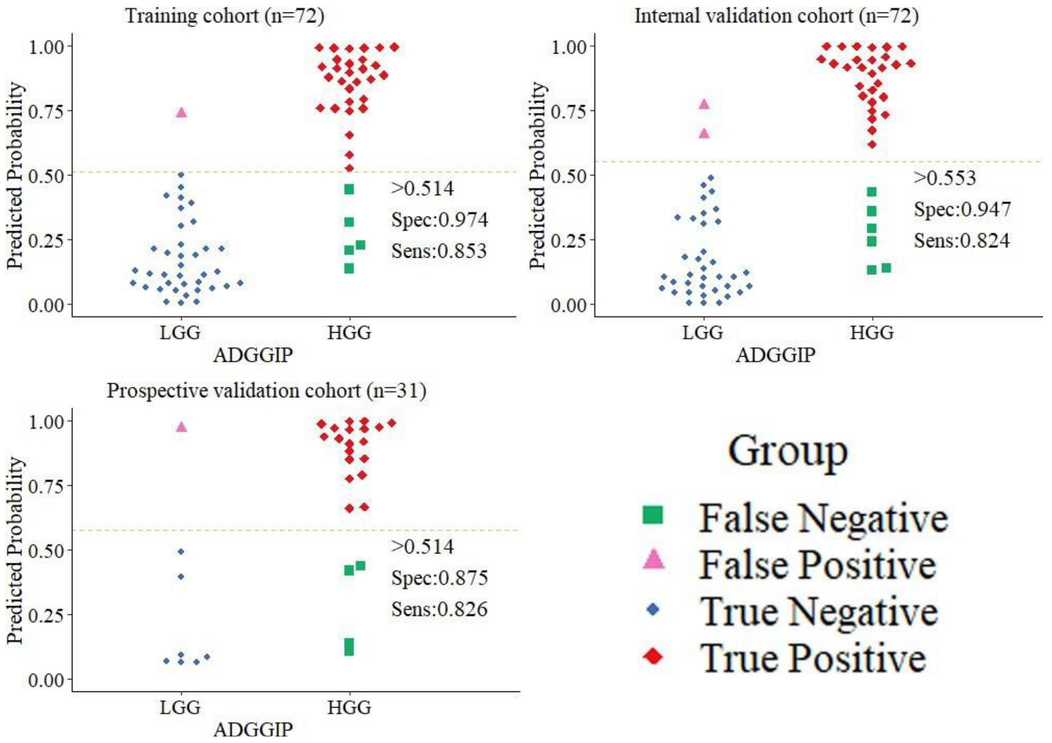

Figure S7. Prediction profiles of ADGGIP

All individual participants were identified as ‘LGG' or ‘HGG' by ADGGIP and assigned into four groups according to their true pathological response in all datasets. Using the optimal risk threshold, the sensitivity and specificity were determined.
LGG = low-grade glioma, HGG = high-grade glioma. Spec = specificity, Sens = sensitivity.

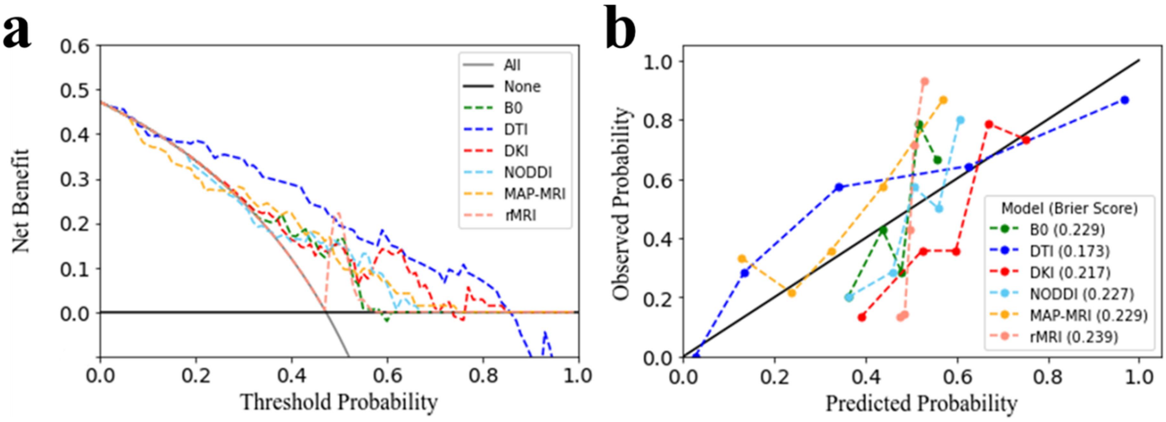

Figure S8. Decision curve analysis (a) and calibration curves (b) of single-modality prediction models in the training cohorts

B0 = diffusion b0 parameter diagram, DTI = diffusion tensor imaging, DKI = diffusion kurtosis imaging, NODDI = neurite orientation dispersion and density imaging, MAP-MRI = mean apparent propagation diffusion magnetic resonance imaging, rMRI = radiomics MRI.

Supplementary Tables

Table S1. Anatomical and diffusion MRI parameters

	**T1**	**T2**	**T2-FLAIR**	**DWI**	**CE-T1**	**DSI**
TA	1 min 46 s	1 min 45 s	2 min 2 s	1 min 4 s	5 min 21 s	15 min 40 s
TR (ms)	1600	5500	6000	3530	2300	7000
TE (ms)	10	117	81	81、128	2.32	107
Slice thickness (mm)	5.5	5.5	5.5	5.5	0.9	3
FOV (mm2)	230*230	230*230	230*230	230*230	240*240	220*220
Base resolution	320	320	320	160	256	98
Phase resolution	75	100	70	100	256	98
Voxel size (mm3)	0.4*0.4*5.5	0.7*0.7*5.5	0.7*0.7*5.5	1.4*1.4*5.5	0.9*0.9*0.9	2.2*2.2*3.0
Diffusion mode	-	-	-	3-Scan Trace	-	q-Space
b-value (s/mm2)	-	-	-	0,1000	-	0-3000

Note: A diffusion spectrum imaging scheme was used, and a total of 128 diffusion sampling were acquired, consisting of 16 b-values (200, 350, 400, 550, 750, 950, 1150, 1500, 1700, 1850, 1900, 2050, 2250, 2450, 2650 and 3000 s/mm^2). The in-plane resolution was 2.65306 mm.
DSI = diffusion spectrum magnetic resonance imaging.

Table S2. Radiomic features extracted by FeAture Explorer

Feature Groups (N)[a]	Feature Names	Feature Groups (N)[a]	Feature Names
First-order statistics[b] (N = 18)	firstorder_10Percentile	GLCM[b] (N = 24)	glcm_Autocorrelation
	firstorder_90Percentile		glcm_ClusterProminence
	firstorder_Energy		glcm_ClusterShade
	firstorder_Entropy		glcm_ClusterTendency
	firstorder_InterquartileRange		glcm_Contrast
	firstorder_Kurtosis		glcm_Correlation
	firstorder_Maximum		glcm_DifferenceAverage
	firstorder_Mean		glcm_DifferenceEntropy
	firstorder_MeanAbsoluteDeviation		glcm_DifferenceVariance
	firstorder_Median		glcm_Id
	firstorder_Minimum		glcm_Idm
	firstorder_Range		glcm_Idmn
	firstorder_RobustMeanAbsoluteDeviation		glcm_Idn
	firstorder_RootMeanSquared		glcm_Imc1
	firstorder_Skewness		glcm_Imc2
	firstorder_TotalEnergy		glcm_InverseVariance
	firstorder_Uniformity		glcm_JointAverage
	firstorder_Variance		glcm_JointEnergy
			glcm_JointEntropy
			glcm_MCC
			glcm_MaximumProbability
			glcm_SumAverage
			glcm_SumEntropy
			glcm_SumSquares
Shape-based[b] (N = 14)	shape_Elongation	GLDM[b] (N = 14)	gldm_DependenceEntropy
	shape_Flatness		gldm_DependenceNonUniformity
	shape_LeastAxisLength		gldm_DependenceNonUniformityNormalized
	shape_MajorAxisLength		gldm_DependenceVariance
	shape_Maximum2DDiameterColumn		gldm_GrayLevelNonUniformity
	shape_Maximum2DDiameterRow		gldm_GrayLevelVariance
	shape_Maximum2DDiameterSlice		gldm_HighGrayLevelEmphasis
	shape_Maximum3DDiameter		gldm_LargeDependenceEmphasis

Shape-based[b] (N = 14)	shape_MeshVolume	GLDM[b] (N = 14)	gldm_LargeDependenceHighGrayLevelEmphasis
	shape_MinorAxisLength		gldm_LargeDependenceLowGrayLevelEmphasis
	shape_Sphericity		gldm_LowGrayLevelEmphasis
	shape_SurfaceArea		gldm_SmallDependenceEmphasis
	shape_SurfaceVolumeRatio		gldm_SmallDependenceHighGrayLevelEmphasis
	shape_VoxelVolume		gldm_SmallDependenceLowGrayLevelEmphasis
GLRLM[b] (N = 16)	glrlm_GrayLevelNonUniformity	GLSZM[b] (N = 16)	glszm_GrayLevelNonUniformity
	glrlm_GrayLevelNonUniformityNormalized		glszm_GrayLevelNonUniformityNormalized
	glrlm_GrayLevelVariance		glszm_GrayLevelVariance
	glrlm_HighGrayLevelRunEmphasis		glszm_HighGrayLevelZoneEmphasis
	glrlm_LongRunEmphasis		glszm_LargeAreaEmphasis
	glrlm_LongRunHighGrayLevelEmphasis		glszm_LargeAreaHighGrayLevelEmphasis
	glrlm_LongRunLowGrayLevelEmphasis		glszm_LargeAreaLowGrayLevelEmphasis
	glrlm_LowGrayLevelRunEmphasis		glszm_LowGrayLevelZoneEmphasis
	glrlm_RunEntropy		glszm_SizeZoneNonUniformity
	glrlm_RunLengthNonUniformity		glszm_SizeZoneNonUniformityNormalized
	glrlm_RunLengthNonUniformityNormalized		glszm_SmallAreaEmphasis
	glrlm_RunPercentage		glszm_SmallAreaHighGrayLevelEmphasis
	glrlm_RunVariance		glszm_SmallAreaLowGrayLevelEmphasis
	glrlm_ShortRunEmphasis		glszm_ZoneEntropy
	glrlm_ShortRunHighGrayLevelEmphasis		glszm_ZonePercentage
	glrlm_ShortRunLowGrayLevelEmphasis		glszm_ZoneVariance
NGTDM[b] (N = 5)	ngtdm_Busyness		
	ngtdm_Coarseness		
	ngtdm_Complexity		
	ngtdm_Contrast		
	ngtdm_Strength		

Note: For the original image, 7 feature types were extracted, and a total of 107 features were obtained.
[a]: The total number of features in a distinct group.
[b]: Included morphological features, first-order histogram features and second-order features.
GLCM = gray level cooccurrence matrix, GLRLM = gray level run length matrix, GLSZM = gray level size zone matrix, GLDM = gray level dependence matrix, NGTDM = neighborhood gray tone difference matrix.

Table S3. Adult-type diffuse gliomas classification proportions among cohorts

Variable	Training and validationcohort (n=72)	Prospective validationcohort (n=31)	P value
**Age(years)[a]**	51.72±11.71	52.55±13.32	.753
**Sex[a]**			.452
Male	36/72 (50.00%)	18/31 (58.06%)	
Female	36/72 (50.00%)	13/31 (41.94%)	
**2021 WHO Integrated Diagnosis (CNS WHO Grade)**			.103
Astrocytoma, IDH-mutant (2)	8/72 (11.11%)	3/31 (9.67%)	
Astrocytoma, IDH-mutant (3)	9/72 (12.50%)	1/31 (3.23%)	
Astrocytoma, IDH-mutant (4)	0/72 (0.00%)	1/31 (3.23%)	
Oligodendroglioma, IDH-mutant, and 1p/19q-codeleted (2)	10/72 (13.89%)	1/31 (3.23%)	
Oligodendroglioma, IDH-mutant, and 1p/19q-codeleted (3)	11/72 (15.28%)	3/31 (9.67%)	
Glioblastoma, IDH-wildtype (4)	34/72 (47.22%)	22/31 (70.97%)	
**Grade**			.012
LGG	38/72 (52.78%)	8/31 (25.81%)	
HGG	34/72 (47.22%)	23/31 (74.19%)	
**Necrosis**			.782
Present	53/72 (73.61%)	22/31 (72.82%)	
Absent	19/72 (26.39%)	9/31 (27.18%)	
**Hemorrhage**			.782
Present	53/72 (73.61%)	22/31 (70.97%)	
Absent	19/72 (26.39%)	9/31 (29.03%)	
**Calcification**			.968
Present	16/72 (22.22%)	7/31 (22.58%)	
Absent	56/72 (77.78%)	24/31 (77.42%)	
**Cyst or cysts**			.940
Present	63/72 (87.50%)	28/31 (90.32%)	
Absent	9/72 (12.50%)	3/31 (9.68%)	
**Edema (≤1.5 cm)**			.843
Yes	41/72 (56.94%)	17/31 (54.84%)	
No	31/72 (43.06%)	14/31 (45.16%)	
**Tumor borders**			.313
Sharp	34/72 (47.22%)	18/31 (58.06%)	
Blurry	38/72 (52.78%)	13/31 (41.94%)	
**Tumor location category**			.011
Frontal or insula	44/72 (61.11%)	9/31 (51.46%)	
Other	4/72 (5.56%)	4/31 (7.77%)	
Basal nucleus or corpus callosum	24/72 (33.33%)	18/31 (40.78%)	
**Side**			.046
Left	41/72 (56.94%)	11/31 (35.48%)	
Right	31/72 (43.06%)	20/31 (64.52%)	
**Enhancement category**			.167
Patchy enhancing	17/72 (23.61%)	8/31 (25.81%)	
Ringlike enhancing	43/72 (59.72%)	22/31 (70.97%)	
Nonenhancing	12/72 (16.67%)	1/31 (3.23%)	

Note: Data are the mean ± SD or n/N (%), where N is the total number of study participants with available data. P values were calculated with the chi-square test, Fisher's test, Student's t test and Mann–Whitney U test.
[a]: Clinical data (age and sex) were obtained from the medical record system or in person.
CNS WHO Grade: Central Nervous System World Health Organization Grade. IDH = isocitrate dehydrogenase, 1p/19q = synchronous deletion of the short arm of chromosome 1 andlong arm of chromosome 19, LGG = low-grade glioma, HGG = high-grade glioma.

Table S4. Prediction performance of single-modality models

	Training cohort	Internal validation cohort	Prospective validation cohort
**B0**			
AUC*	0.721 (0.591–0.832)	0.773 (0.664–0.869)	0.717 (0.495–0.902)
Sensitivity	0.588 (20/34)	0.765 (26/34)	0.739 (17/23)
Specificity	0.816 (31/38)	0.737 (28/38)	0.500 (4/8)
PPV	0.741 (20/27)	0.722 (26/36)	0.810 (17/21)
NPV	0.689 (31/45)	0.778 (28/36)	0.400 (4/10)
ACC	0.708 (51/72)	0.750 (54/72)	0.677 (21/31)
**DTI**			
AUC*	0.851 (0.767–0.930)	0.821 (0.723–0.909)	0.832 (0.663–0.962)
Sensitivity	0.735 (25/34)	0.853 (29/34)	0.783 (18/23)
Specificity	0.816 (31/38)	0.711 (27/38)	0.625 (5/8)
PPV	0.781 (25/32)	0.725 (29/40)	0.857 (18/21)
NPV	0.775 (31/40)	0.844 (27/32)	0.500 (5/10)
ACC	0.778 (56/72)	0.778 (56/72)	0.742 (23/31)
**DKI**			
AUC*	0.780 (0.664–0.876)	0.756 (0.646–0.858)	0.766 (0.576–0.929)
Sensitivity	0.647 (22/34)	0.706 (24/34)	0.696 (16/23)
Specificity	0.816 (31/38)	0.737 (28/38)	0.750 (6/8)
PPV	0.759 (22/29)	0.706 (24/34)	0.889 (16/18)
NPV	0.721 (31/43)	0.737 (28/38)	0.462 (6/13)
ACC	0.736 (53/72)	0.722 (52/72)	0.710 (22/31)
**NODDI**			
AUC*	0.732 (0.632–0.851)	0.709 (0.587–0.831)	0.734 (0.554–0.902)
Sensitivity	0.677 (23/34)	0.588 (20/34)	0.478 (11/23)
Specificity	0.711 (27/38)	0.816 (31/38)	0.875 (7/8)
PPV	0.677 (23/34)	0.741 (20/27)	0.917 (11/12)
NPV	0.711 (27/38)	0.689 (31/45)	0.368 (7/19)
ACC	0.694 (50/72)	0.708 (51/72)	0.581 (18/31)
**MAP-MRI**			
AUC	0.721 (0.594–0.840)	0.731 (0.611–0.843)	0.777 (0.587–0.929)
Sensitivity	0.559 (19/34)	0.824 (28/34)	0.522 (12/23)
Specificity	0.842 (32/38)	0.658 (25/38)	0.750 (6/8)
PPV	0.760 (19/25)	0.683 (28/41)	0.857 (12/14)
NPV	0.681 (32/47)	0.807 (25/31)	0.353 (6/17)
ACC	0.708 (51/72)	0.736 (53/72)	0.581 (18/31)

Note: Data in parentheses are the numerator/denominator of participants included for each parameter, unless otherwise indicated. Values correspond to the optimal threshold according to the maximum Youden index.

*: Data are the mean (95% CI).

AUC = area under the curve, PPV =positive predictive value, NPV = negative predictive value, ACC = accuracy. B0 = diffusion b0 parameter diagram, DTI = diffusion tensor imaging, DKI =diffusion kurtosis imaging, NODDI = neurite orientation dispersion and density imaging, MAP-MRI = mean apparent propagation diffusion magnetic resonance imaging.

Tal le S5. Delong test for ROC curve improvements n multiple coho rts

Model	Training cohort		Internal validation cohort		Prospective validation cohort	
	Difference between areas (95% CI)	P value	Difference between areas (95% CI)	P value	Difference between areas (95% CI)	P value
ADGGIP	/	/	/	/	/	/
CliRadDTI	0.046 (-0.004–0.096)	.074	0.452 (0.206 – 0.697)	<.001*	0.044 (-0.128 – 0.215)	.620
ClinicRad	0.118 (0.031–0.204)	.008*	0.387 (0.179 – 0.594)	<.001*	0.044 (-0.086 – 0.173)	.512
rMRI	0.098 (0.014–0.183)	.023*	0.467 (0.223 – 0.711)	<.001*	0.011 (-0.124 – 0.146)	.874
MAP-MRI	0.237 (0.108–0.365)	<.001*	0.285 (0.083 – 0.488)	.006*	0.103 (-0.054 – 0.261)	.199
NODDI	0.226 (0.103–0.349)	<.001*	0.434 (0.157 – 0.712)	.002*	0.147 (-0.124 – 0.418)	.289
DKI	0.178 (0.067–0.290)	.002*	0.385 (0.171 – 0.599)	<.001*	0.114(-0.024 – 0.252)	.105
DTI	0.108 (0.017–0.198)	.020*	0.116 (-0.169 – 0.400)	.426	0.049 (-0.153 – 0.251)	.635
B0	0.238 (0.112–0.363)	<.001*	0.453 (0.232 – 0.673)	<.001*	0.163 (0.026 – 0.300)	.020*
rMRI	/	/	/	/	/	/
B0	0.139 (0.023–0.255)	.019*	-0.014 (-0.091 – 0.062)	.712	0.152 (-0.037 – 0.342)	.116
DTI	0.009 (-0.046–0.064)	.741	-0.352 (-0.598 – -0.105)	.005*	0.038 (-0.075 – 0.151)	.510
DKI	0.080 (-0.020–0.180)	.119	-0.082 (-0.182 – 0.018)	.108	0.103 (-0.030 – 0.236)	.128
NODDI	0.128 (-0.023–0.279)	.097	-0.033 (-0.114 – 0.049)	.433	0.136 (-0.081 – 0.353)	.219
MAP-MRI	0.139 (0.001–0.276)	.048*	-0.182 (-0.323 – -0.040)	.012*	0.092 (-0.093 – 0.278)	.329
DTI	/	/	/	/	/	/
B0	0.130 (0.015–0.246)	.027*	0.337 (0.106 – 0.569)	.004*	0.114 (-0.037 – 0.342)	.327
DKI	0.070 (-0.043–0.184)	.223	0.270 (0.074 – 0.465)	.007*	0.065 (-0.096 – 0.227)	.429
NODDI	0.118 (-0.044–0.281)	.153	0.319 (0.034 – 0.604)	.028*	0.098 (-0.151 – 0.347)	.441
MAP-MRI	0.129 (-0.002–0.260)	.053	0.170 (-0.137 – 0.477)	.279	0.054 (-0.182 – 0.290)	.652

*: P <. 05 indicated a significant difference between models.
B0 = diffusion b0 parameter diagram, DTI = diffusion tensor imaging, DKI = diffusion kurtosis imaging, NODDI = neurite orientation dispersion and density imaging, MAP-MRI = mean apparent propagation diffusion magnetic resonance imaging. rMRI = radiomics MRI, ClinicRad = model incorporating clinical factors and interpretations from radiologists, CliRadDTI = model incorporating clinical factors, radiologist interpretations and DTI data, ADGGIP = Adult-type Diffuse Gliomas Grade Integrated Prediction model.

Table S6. IDI test for prediction improvements in multiple cohorts

Model	Training cohort		Internal validation cohort		Prospective validation cohort	
	IDI (95% CI)	P value	IDI (95% CI)	P value	IDI (95% CI)	P value
ADGGIP	/	/	/	/	/	/
CliRadDTI	0.520 (0.430 – 0.610)	<.001*	0.508 (0.410 – 0.607)	<.001*	0.452 (0.206 – 0.697)	<.001*
ClinicRad	0.491 (0.404 – 0.578)	<.001*	0.485 (0.391 – 0.580)	<.001*	0.387 (0.179 – 0.594)	<.001*
rMRI	0.567 (0.473 – 0.661)	<.001*	0.556 (0.451 – 0.661)	<.001*	0.467 (0.223 – 0.711)	<.001*
MAP-MRI	0.460 (0.341 – 0.579)	<.001*	0.435 (0.315 – 0.554)	<.001*	0.285 (0.083 – 0.488)	.006*
NODDI	0.526 (0.422 – 0.630)	<.001*	0.491 (0.362 – 0.621)	<.001*	0.434 (0.157 – 0.712)	.002*
DKI	0.464 (0.362 – 0.567)	<.001*	0.413 (0.296 – 0.530)	<.001*	0.385 (0.171 – 0.599)	<.001*
DTI	0.182 (0.040 – 0.324)	.012*	0.208 (0.064 – 0.352)	.005*	0.116 (-0.169 – 0.400)	.426
B0	0.538 (0.442 – 0.634)	<.001*	0.517 (0.413 – 0.621)	<.001*	0.453 (0.232 – 0.673)	<.001*
rMRI	/	/	/	/	/	/
B0	-0.029 (0.058 – 0.001)	.052	-0.039 (-0.066 – -0.012)	.005*	-0.014 (-0.091 – 0.062)	.712
DTI	-0.385 (-0.515 – -0.254)	<.001*	-0.348 (-0.481 – -0.216)	<.001*	-0.352 (-0.598 – -0.105)	.005*
DKI	-0.103 (-0.153 – -0.053)	<.001*	-0.143 (-0.226 – -0.061)	<.001*	-0.082 (-0.182 – 0.018)	.108
NODDI	-0.041 (-0.082 – 0.001)	.055	-0.065 (-0.125 – -0.005)	.033*	-0.033 (-0.114 – 0.049)	.433
MAP-MRI	-0.107 (-0.177 – -0.037)	.003*	-0.121 (-0.198 – -0.045)	.002*	-0.182 (-0.323 – -0.040)	.012*
DTI	/	/	/	/	/	/
B0	0.356 (0.227 – 0.485)	<.001*	0.309 (0.169 – 0.449)	<.001*	0.337 (0.106 – 0.569)	.004*
DKI	0.282 (0.152 – 0.412)	<.001*	0.205 (0.063 – 0.346)	.005*	0.270 (0.074 – 0.465)	.007*
NODDI	0.344 (0.194 – 0.494)	<.001*	0.283 (0.139 – 0.427)	<.001*	0.319 (0.034 – 0.604)	.028*
MAP-MRI	0.278 (0.141 – 0.414)	<.001*	0.227 (0.080 – 0.374)	.002*	0.170 (-0.137 – 0.477)	.279

*: P <. 05 indicated a significant difference between models.
IDI = integrated discrimination improvement. B0 = diffusion b0 parameter diagram, DTI = diffusion tensor imaging, DKI = diffusion kurtosis imaging, NODDI = neurite orientation dispersion and density imaging, MAP-MRI = mean apparent propagation diffusion magnetic resonance imaging. rMRI = radiomics MRI, ClinicRad = model incorporating clinical factors and interpretations from radiologists, CliRadDTI = model incorporating clinical factors, radiologist interpretations and DTI data, ADGGIP = Adult-type Diffuse Gliomas Grade Integrated Prediction model.

Table S7. NRI test for prediction improvements in multiple cohorts

Model	Training cohort		Internal validation cohort		Prospective validation cohort	
	NRI (95% CI)	P value	NRI (95% CI)	P value	NRI (95% CI)	P value
ADGGIP	/	/	/	/	/	/
CliRadDTI	1.489 (1.222 – 1.756)	<.001*	1.433 (1.150 – 1.717)	<.001*	1.277 (0.730 – 1.825)	<.001*
ClinicRad	1.101 (0.843 – 1.358)	<.001*	1.098 (0.824 – 1.371)	<.001*	0.777 (0.288 – 1.267)	.002*
rMRI	1.489 (1.224 – 1.756)	<.001*	1.433 (1.150 – 1.717)	<.001*	1.234 (0.685 – 1.783)	<.001*
MAP-MRI	0.791 (0.551 – 1.031)	<.001*	0.808 (0.552 – 1.064)	<.001*	0.571 (0.274 – 0.867)	<.001*
NODDI	1.334 (1.055 – 1.614)	<.001*	1.079 (0.759 – 1.398)	<.001*	0.859 (0.345 – 1.373)	.001*
DKI	1.122 (0.839 – 1.405)	<.001*	0.807 (0.500 – 1.113)	<.001*	0.761 (0.335 – 1.187)	<.001*
DTI	0.429 (0.152 – 0.706)	.002*	0.399 (0.125 – 0.674)	.004*	0.000 (-0.533 – 0.533)	.999
B0	1.255 (0.961 – 1.550)	<.001*	1.226 (0.939 – 1.513)	<.001*	1.152 (0.598 – 1.706)	<.001*
rMRI	/	/	/	/	/	/
B0	-0.175 (-0.344 – -0.006)	.043*	-0.207 (-0.354 – -0.061)	.006*	-0.082 (-0.325 – 0.162)	.512
DTI	-0.920 (-1.261 – -0.578)	<.001*	-0.864 (-1.214 – -0.514)	<.001*	-1.022 (-1.617 – -0.426)	<.001*
DKI	-0.317 (-0.552 – -0.083)	.008*	-0.598 (-0.868 – -0.327)	<.001*	-0.429 (-0.901 – 0.042)	.074
NODDI	-0.125 (-0.272 – -0.021)	.093	-0.240 (-0.453 – -0.027)	.027*	-0.370 (-0.780 – 0.040)	.077
MAP-MRI	-0.460 (-0.676 – -0.244)	<.001*	-0.475 (-0.748 – -0.203)	<.001*	-0.402 (-0.816 – 0.012)	.057
DTI	/	/	/	/	/	/
B0	0.742 (0.393 – 1.090)	<.001*	0.683 (0.329 – 1.036)	<.001*	0.940 (0.352 – 1.529)	.002*
DKI	0.605 (0.271 – 0.940)	<.001*	0.322 (-0.042 – 0.686)	.083	0.549 (-0.025 – 1.123)	.061
NODDI	0.762 (0.415 – 1.108)	<.001*	0.624 (0.264 – 0.983)	<.001*	0.690 (-0.015 – 1.395)	.055
MAP-MRI	0.336 (0.088 – 0.584)	.008*	0.297 (-0.018 – 0.612)	.064	0.397 (-0.097 – 0.890)	.115

Note: Cutoff values for risk categories. Define the cutoff values as (0, 0.4, 0.7, 1).

*: P <. 05 indicated a significant difference between models.

NRI = net reclassification improvement. B0 = diffusion b0 parameter diagram, DTI = diffusion tensor imaging, DKI = diffusion kurtosis imaging, NODDI = neurite orientation dispersion and density imaging, MAP-MRI = mean apparent propagation diffusion magnetic resonance imaging. rMRI = radiomics MRI, ClinicRad = model incorporating clinical factors and interpretations from radiologists, CliRadDTI = model incorporating clinical factors, radiologist interpretations and DTI data, ADGGIP = Adult-type Diffuse Gliomas Grade Integrated Prediction model.

Table S8. Brier score for ADGGIP in achieving prediction improvements compared with other models in the training cohort

**Model**	**Brier score**
ADGGIP	0.084
CliRadDTI	0.221
ClinicRad	0.208
rMRI	0.239
MAP-MRI	0.229
NODDI	0.227
DKI	0.217
DTI	0.173
B0	0.229

B0 = diffusion b0 parameter diagram, DTI = diffusion tensor imaging, DKI = diffusion kurtosis imaging, NODDI = neurite orientation dispersion and density imaging, MAP-MRI = mean apparent propagation diffusion magnetic resonance imaging. rMRI = radiomics MRI, ClinicRad = model incorporating clinical factors and interpretations from radiologists, CliRadDTI = model incorporating clinical factors, radiologist interpretations and DTI data, ADGGIP = Adult-type Diffuse Gliomas Grade Integrated Prediction model.
